# Seaweed-Derived Phlorotannins: A Review of Multiple Biological Roles and Action Mechanisms

**DOI:** 10.3390/md20060384

**Published:** 2022-06-08

**Authors:** Fazlurrahman Khan, Geum-Jae Jeong, Mohd Sajjad Ahmad Khan, Nazia Tabassum, Young-Mog Kim

**Affiliations:** 1 Research Center for Marine Integrated Bionics Technology, Pukyong National University, Busan 48513, Korea; 2 Department of Food Science and Technology, Pukyong National University, Busan 48513, Korea; jgj1994@pukyong.ac.kr; 3 Department of Basic Sciences, Deanship of Preparatory Year and Supporting Studies, Imam Abdulrahman Bin Faisal University, P.O. Box 1982, Dammam 34212, Saudi Arabia; mskhan@iau.edu.sa; 4 Industry 4.0 Convergence Bionics Engineering, Pukyong National University, Busan 48513, Korea; nazia99@pukyong.ac.kr

**Keywords:** marine organisms, seaweeds, phlorotannins, antimicrobial, antioxidant, antidiabetic

## Abstract

Phlorotannins are a group of phenolic secondary metabolites isolated from a variety of brown algal species belonging to the Fucaceae, Sargassaceae, and Alariaceae families. The isolation of phlorotannins from various algal species has received a lot of interest owing to the fact that they have a range of biological features and are very biocompatible in their applications. Phlorotannins have a wide range of therapeutic biological actions, including antimicrobial, antidiabetic, antioxidant, anticancer, anti-inflammatory, anti-adipogenesis, and numerous other biomedical applications. The current review has extensively addressed the application of phlorotannins, which have been extensively investigated for the above-mentioned biological action and the underlying mechanism of action. Furthermore, the current review offers many ways to use phlorotannins to avoid certain downsides, such as low stability. This review article will assist the scientific community in investigating the greater biological significance of phlorotannins and developing innovative techniques for treating both infectious and non-infectious diseases in humans.

## 1. Introduction

**Figure 1:**
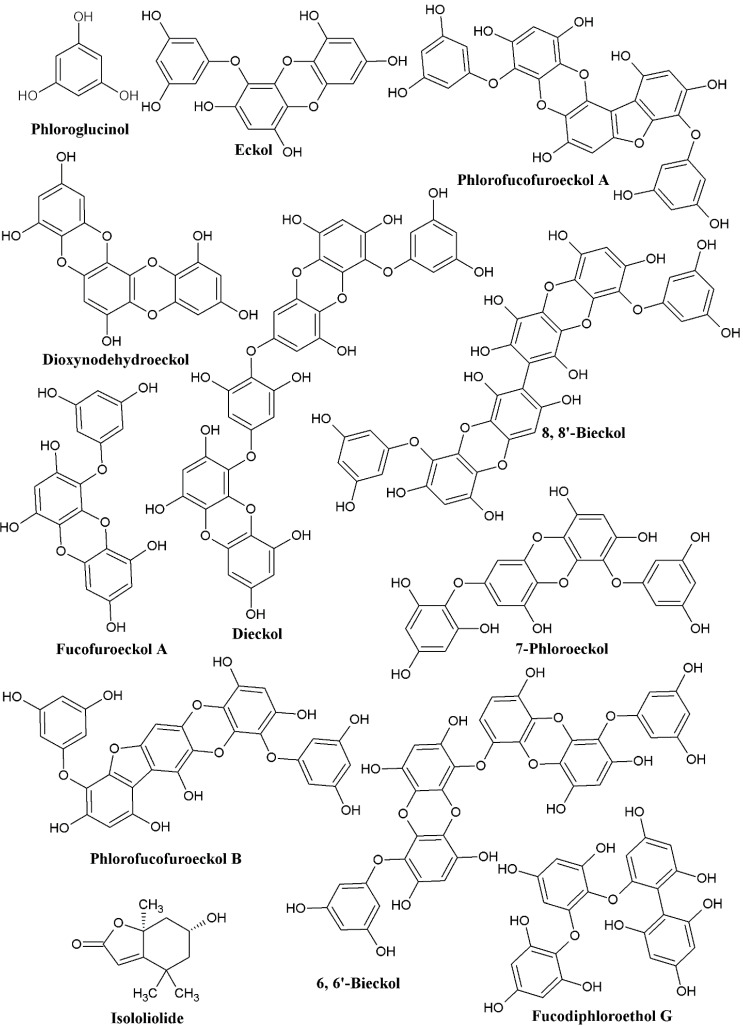
Chemical structures of phlorotannins.

Marine organisms, particularly brown seaweeds, have garnered a lot of interest across the world for their potential use in treating a variety of infectious and non-infectious diseases [1,2]. Several compounds have been isolated from brown seaweeds, including phlorotannins, fucoxanthin, fucoidan, alginate, fucoidan, and laminarin [3]. Almost all secondary metabolites have been found to possess various biological activities. Among these, phlorotannins, which are members of the polyphenolic group of secondary metabolites, has been found in abundance in brown algae belonging to the Fucaceae, Sargassaceae, and Alariaceae families [4]. Some red and green algae also contain phlorotannins, albeit to a lesser extent than brown algae [5]. Despite problems in isolating and purifying phlorotannins from brown seaweeds, more than 150 phlorotannins have been identified [6]. The different sources from which phlorotannins have been isolated and identified are given in Table 1 and Table 2. Figure 1 depicts the chemical structures of these phlorotannins. The use of traditional antibiotics and other pharmaceuticals to treat infectious diseases caused by bacterial, viral, and fungal pathogens [7,8,9], as well as non-infectious diseases such as cancer, diabetes, inflammation, obesity, and adipogenesis, has been shown to be detrimental [10,11,12]. Microbial infections caused by viruses, bacteria, and fungus have become one of the most serious threats to human health across the world [13,14,15].

Furthermore, microbial pathogens such as bacteria and fungi evolved many resistance mechanisms against commonly used antibiotics and antifungals [16,17]. Multi-drug resistant strains of microorganisms from various hospital and clinical environments are also rapidly growing [18,19,20]. With rising antimicrobial resistance, the characterization of microbial pathogens and innovative strategies to combat microbial diseases are in great demand. Recent and previous research has found that phlorotannins have antibacterial, antifungal, and antiviral effects against a wide variety of microbial pathogens [21,22,23]. Non-infectious disorders, including cancer, diabetes, inflammation, and obesity, are also on the rise and creating a strong need for newer therapeutic drugs [24,25,26]. The current review addresses the biological properties of several types of phlorotannins in treating both infectious and non-infectious diseases. Furthermore, the underlying mechanism involved in treating diseases using phlorotannins have also been explored. Additionally, the current limitations and future perspectives associated with the isolation and application of phlorotannins have been thoroughly discussed.

## 2. Phlorotannin against Pathogenic Bacterial and Fungal Species

**Figure 2:**
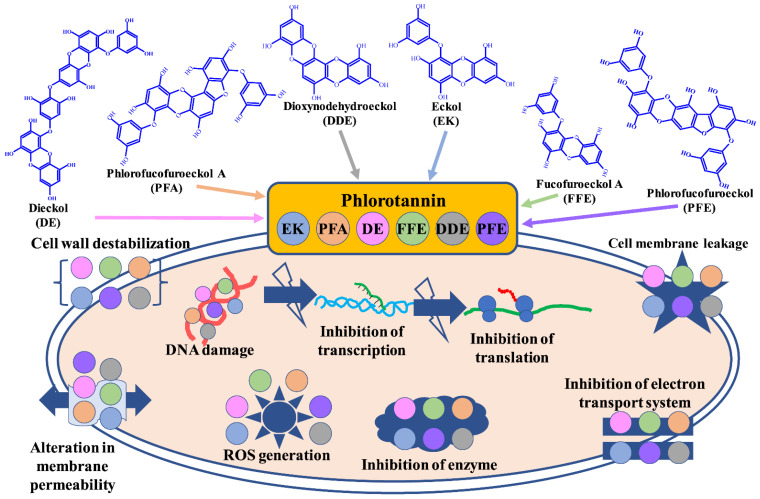
Mechanism of antibacterial and antifungal activities of different types of phlorotannins.

Antibiotics are used to treat the majority of bacterial pathogens, but the development of antibiotic resistance in pathogens has recently grown substantially, directly affecting human mortality. Antibiotic resistance is mostly caused by the indiscriminate use of antibiotics for pathogen treatment, long-term antibiotic therapy for patients, and patient discontinuation of antibiotics before finishing treatment [27]. According to reports, *Acinetobacter baumannii, Enterococcus faecium*, *Enterobacter* spp., *Klebsiella pneumoniae, Pseudomonas aeruginosa*, and *Staphylococcus* spp. are the major bacterial pathogens involved in the development of antibiotic resistance [28]. Antibiotic resistance can be developed or acquired through processes such as (1) lower intracellular concentration of antibiotics owing to pathogen efflux, (2) genetic change of the antibiotic target, and (3) antibiotic deactivation by hydrolysis or modification [29]. This is comparable to the situation in fungal pathogens, where studies have shown that antifungal drugs are successful in the treatment of common fungal pathogens such as *Candida* spp., *Cryptococcus* spp., and *Aspergillus* spp. However, fungi develop resistance as a result of the indiscriminate use of antifungal drugs, resulting in increased fungal disease mortality [30]. Furthermore, the majority of antifungal agents utilized in the treatment of human diseases include polyenes, triazoles, and echinocandins, but they are toxic to patients [31]. Aside from intrinsic and acquired resistance mechanisms, bacterial and fungal species have evolved phenotypic adaptive resistance mechanisms known as biofilm formation, which is an assembly of cells surrounded by self-produced polymeric substances [32]. Biofilms act as a structural and functional barrier to the entry of antibacterial and antifungal drugs into the microbial cell. Furthermore, these biofilms contained a small population of cells (persister cells) that are metabolically inactive and have been linked to persistent and recurring infection. As a result, there is an urgent need to develop a strategy or find novel molecules to battle antibiotic-resistant bacteria and fungi. Several studies have proven the antibacterial action of phlorotannins isolated from various seaweeds. Phlorotannins have an antibacterial effect by suppressing oxidative phosphorylation, altering target microorganism cell permeability, and triggering death by interacting with target microbial proteins [33]. The phenolic aromatic ring and hydroxyl groups of various phlorotannins have been shown to bind to microbial proteins and induce cell lysis [34]. The susceptibility of Gram-positive and Gram-negative bacteria to phlorotannins varies owing to structural differences in the cell membrane and cell wall. Gram-negative bacteria are often less susceptible to phlorotannin than Gram-positive bacteria [35]. Nagayama et al. [21] exhibited the bactericidal action of phlorotannins derived from *Ecklonia kurome* for the first time, where phlorotannins showed excellent activity against MRSA. Several types of phlorotannins isolated from *Eisenia bicyclis* showed antibacterial activity against *Listeria monocytogenes* at 16–256 µg/mL [36]. Furthermore, the combination of phlorotannins and streptomycin demonstrated strong synergistic activity against aminoglycoside antibiotic-resistant clinical strains of *L. monocytogenes* (fractional inhibitory concentration index, ∑FIC < 0.5). Cox et al. [37] discovered that extracts prepared from several seaweeds inhibited *E. faecalis, L. monocytogenes*, *P. aeruginosa*, and *Salmonella abony*. Brown seaweed, for instance, outperformed red and green seaweed in antibacterial activity. Another research group found that phlorotannins inhibited quorum sensing (QS) activity in *Chromobacterium violaceum* 12472 by preventing the synthesis of purple pigment [38]. Similarly, phlorotannins reduced *P. aeruginosa* virulence by inhibiting motility and decreasing the production of virulence factors such as extracellular protease, hemolysis, and pyocyanin. Eom et al. [39] conducted the first investigation on the anti-MRSA (methicillin-resistant *S. aureus*) activity of phlorotannins from *E. bicyclis*. The anti-MRSA activity of phlorofucofuroeckol-A was associated with a MIC value of 32 µg/mL. Furthermore, the combinations of phlorofucofuroeckol-A and β-lactam antibiotics such as ampicillin, penicillin, and oxacillin have shown strong synergistic effects. Choi et al. [40] discovered phlorotannin activity against the acne-causing bacteria *Propionibacterium acnes*. Dieckol and phlorofucofuroeckol-A, in particular, were found to be very active against acne-causing bacteria when isolated from *E. cava*. Lee et al. [41] also discovered that the compounds isolated from *E. bicyclis* have strong antibacterial action against acne-causing bacteria. Furthermore, fucofuroeckol-A combined with erythromycin and lincomycin had a synergistic impact on *P. acnes*. Ethanolic extracts of *E. cava* were found to have effective antibacterial activity towards *S. aureus*, MRSA, and *Salmonella* spp. [42]. Eckol from *E. cava* substantially reduced the MIC value of ampicillin against MRSA and *Salmonella* spp. Mittal et al. [43] discovered that phloroglucinol and related compounds exhibited antibacterial action against various Gram-positive bacteria. Furthermore, the combination of phlorodipropanophenone and oxycycline had a strong synergistic bactericidal effect. Kim et al. assessed the antibacterial activity of *E. cava* phlorotannin against fish infectious diseases caused by marine bacterial pathogens [44]. The methanol extract of *E. cava* was shown to have potent antibacterial action against several pathogens such as *Streptococcus iniae*, *Edwardsiella tarda*, *S. parauberis*, *V. harveyi, V. scophthalm*, and *Vibrio anguillarum*. Phlorotannin isolated from *E. arborea* has shown antibacterial action against *V. parahaemolyticus*, indicating its potential as an antibiotic to combat acute hepatopancreatic necrosis disease caused by this pathogen [45]. Furthermore, dieckol derived from *E. stolonifera* has shown antibacterial efficacy against MRSA and methicillin-susceptible *S. aureus* (MSSA) [46]. In addition, the application of dieckol reversed the resistance properties developed against ampicillin and penicillin by MRSA. Wei et al. [47] studied the mechanism of phlorotannins derived from *Sargassum thunbergii* against *V. parahaemolyticus*. Their findings revealed that phlorotannin was able to suppress bacterial growth and damaged the cytoplasmic membrane. Some studies have also found that phlorotannins damage the cell membranes of anaerobic bacteria, resulting in extracellular and intracellular repercussions [48]. According to Lopes et al. [22] phlorotannin isolated from seaweed has an inhibitory effect against yeast and dermatophytes. Dieckol extracted from *E. cava* showed fungicidal activity towards *Trichophyton rubrum* (200 μM) [49], resulting in the loss of cytoplasmic membrane integrity. According to Kim et al. [50] fucofuroeckol-A has significant antifungal action towards *C. albicans* with the MIC value of 512 μg/mL. Fucofuroeckol-A enhanced fluconazole’s antifungal activity against fluconazole-resistant *C. albicans*. Figure 2 depicts a diagrammatic explanation of the mechanism involved in the antimicrobial activity of several forms of phlorotannins against bacterial and fungal pathogens. Table 1 shows several other examples of phlorotannins with antibacterial and antifungal activities. Table 1 summarizes a number of additional phlorotannins having potent antimicrobial activity.

## 3. Phlorotannins as a Natural Adjuvant of Antiviral Therapy

**Figure 3:**
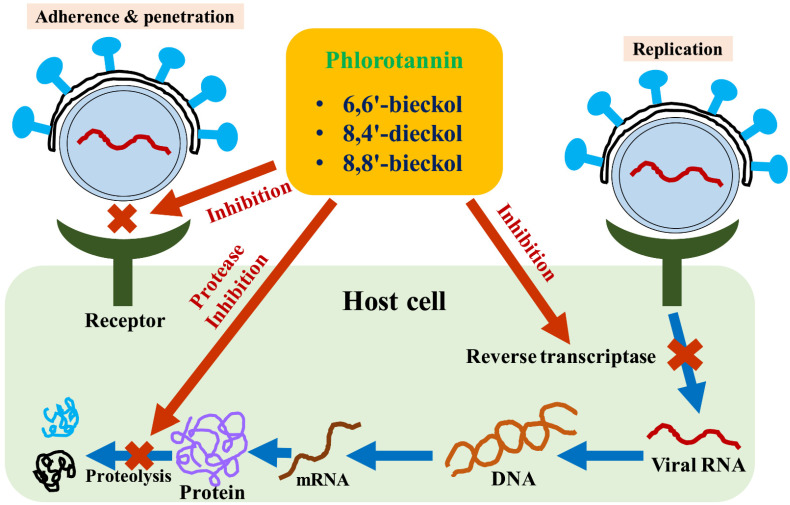
Mechanism of antiviral activities of phlorotannins.

Viral diseases are one of the major issues caused by several viruses, including the human immunodeficiency virus [51], hepatitis B and C virus, and coronavirus (COVID-19). All of these have a severe negative impact on human life. Antiviral agents exhibit a variety of action mechanisms, including inhibition of viral fusion and mRNA entry, antagonism of chemokine co-receptors and cyclophilins, and inhibition of proteases and reverse transcriptase within the host cell (Figure 3). Viruses are acellular and have a simple structure with a protein coat, nucleic acid, viral enzyme, and a lipid envelope, where they replicate after infecting host cells [52]. The viral life cycle inside host cells is extremely complicated, requiring several phases from invasion through maturity. As a result, one of the key problems in designing antiviral agents with selective toxicity is the lack of these characteristics [53]. Although various antiviral medications have been identified, the existing antiviral agents have significant drawbacks, such as the development of resistance, limited bioavailability, and high toxicity [54]. Besednova et al. [55] demonstrated that compounds with antiviral activities obtained from marine species do not cause resistance owing to a variety of mechanisms, including antiviral, immunostimulatory, anti-inflammatory, and antioxidant capabilities. One of the compounds obtained from marine organisms, phlorotannin, has high antioxidant capabilities, particularly due to the presence of the phenol group. These properties have been shown to prevent viral infection as well as interfere with virus adherence, penetration, and replication [56]. According to Artan et al. [57], 6,6′-bieckol derived from *E. cava* prevented HIV-1 induced fusion formation (EC_50_ 1.72 μM), viral p24 antigen production (EC_50_ 1.26 μM), and lytic effect (EC_50_ 1.23 μM), thereby exhibiting antiviral activities. Moreover, at concentrations that suppressed HIV-1 replication, 6,6′-bieckol did not exhibit cytotoxicity. At non-cytotoxic doses, 8, 4′′′-dieckol derived from *E. cava* also inhibited HIV-1 lytic effect and viral p24 antigen production [57]. According to the findings, 8,4′′′-dieckol preferentially inhibited the reverse transcriptase activity of HIV-1 (Figure 3). Ahn et al. [58] discovered that 8,4”-dieckol and 8,8′-bieckol inhibited HIV-1 protease and reverse transcriptase. Furthermore, these drugs inhibited HIV-1 reverse transcriptase’s RNA-dependent DNA synthesis activity against dUTP/dTTP in a non-competitive manner. In a cell-free-based system, phlorotannins from *E. cava* competitively suppressed SARS-CoV-2 3CLpro [23]. These compounds showed an inhibitory effect on 3CLpro hydrolysis (IC_50_ 2.7~164.7 μM). Ryu et al. [59] showed that the ethanol extract of *E. cava* exhibited inhibitory activity against neuraminidase action, thereby inhibiting the influenza virus. In particular, the ethanol extract effectively inhibited neuraminidase by synergizing with oseltamivir.

## 4. Anti-Inflammatory and Immunomodulatory Properties of Phlorotannins

**Figure 4:**
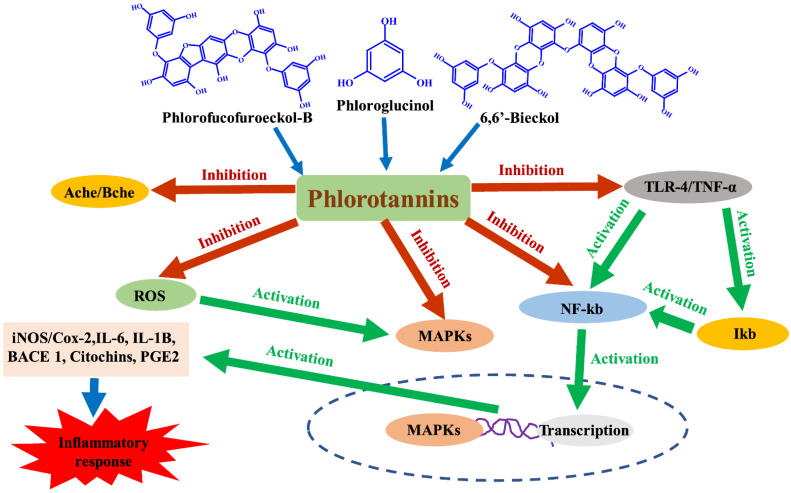
Mechanisms of anti-inflammatory activity of several types of phlorotannins.

Inflammation is the body’s reaction to harmful stimuli, such as an infection or injury, which leads to the development of symptoms such as seizure, pain, fever, and swelling. The inflammatory response is an immunological reaction to exogenous and endogenous signals that serve as the host’s defensive systems against potentially harmful stimuli [32]. An inflammatory response is necessary for survival, and it plays an important role in cellular physiology; nevertheless, uncontrolled and excessive inflammation leads to a variety of chronic diseases. [60]. Inflammatory drugs include both steroidal and nonsteroidal, which are synthetic drugs used to treat inflammatory disorders that have potentially dangerous side effects and are not safe for humans [61]. In recent years, many investigations on the isolation and identification of natural anti-inflammatory drugs have been carried out. As a result, naturally occurring organisms have components that chemically resemble steroid structures and have been reported to exhibit a high potential for anti-inflammatory action [62]. Natural compounds with anti-inflammatory potential have antioxidant and radical scavenging characteristics, as well as the capacity to control the activity of pro-inflammatory enzymes such as phospholipases A2 (PLA2s), cyclooxygenase, and lipoxygenases (LOX) [37]. They also control the production of pro-inflammatory factors and genes [63]. As a result, these discoveries may open the path for the discovery of natural anti-inflammatory drugs. Fucosterol isolated from *E. bicyclis* reduced LPS-induced nitric oxide (NO) generation at non-toxic doses [64]. Furthermore, fucosterol inhibited tert-butyl hydroperoxide-induced reactive oxygen species (ROS) production and the expression of COX-2i and NOS. Several researchers used RAW 264.7 cells activated by lipopolysaccharide (LPS) to assess anti-inflammatory activity. Phlorotannins isolated from Fucales were found to be non-toxic at concentrations ranging from 31.25–500 μg/mL [65]. In RAW 264.7 cells activated by LPS, phloroglucinol reduced the generation of inflammatory mediators such as interleukin-6 (IL-6), interleukin-1β (IL-1β), tumor necrosis factor-α (TNF-α), and prostaglandin E_2_ (PGE_2_) in RAW 264.7 cells stimulated by LPS [66]. Furthermore, phloroglucinol inhibited the production of metalloproteinase, which is involved in chronic inflammation in HT 1080 cells. In macrophages activated by LPS, phlorofucofuroeckol-B isolated from *Myagropsis myagroides* blocked the NF-κB pathway via suppression of the Akt-ERK pathway (Figure 4) [67]. Gonçalves-Fernández et al. [68] confirmed the cytotoxic activity against murine cell lines by identifying fractions of low molecular size and medium polarity among fractionated phlorotannins. At a concentration of 100 µM, the fraction reduced ATDC-5 cell proliferation by 50%. In another study, phlorotannins isolated from the brown macroalga *Padina tetrastromatica* were shown to have strong anti-MRSA, anti-inflammatory, and antioxidant activities [69]. It was discovered, in particular, that the content and structure of phlorotannins differed depending on environmental and geographic conditions. Yu et al. [70] discovered that phlorofucofuroeckol-B isolated from *E. stolonifera* suppressed IL-1β, IL-6, and TNF-α (Figure 4). Furthermore, phlorofucofuroeckol-B suppressed the generation of NO and prostaglandins and downregulated the LPS-induced expression of inducible cyclooxygenase-2 and NO synthase in LPS-stimulated BV-2. The compound 6,6′-Bieckol decreased nitric oxide and prostaglandin production and suppressed LPS-induced expression of inducible cyclooxygenase-2 and NO synthase (Figure 4) [71]. Importantly, 6,6′-bieckol inhibited the expression of IL-6 and TNF-α.

## 5. Phlorotaninns Having Anticancer Potential

**Figure 5:**
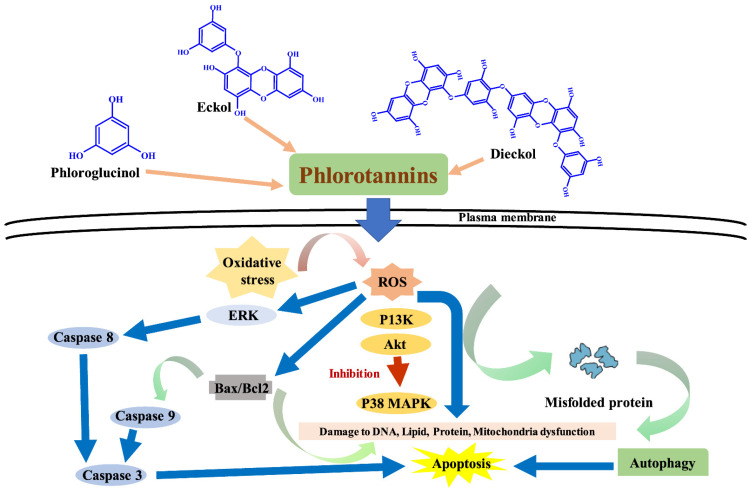
The anticancer activities of different types of phlorotannins.

Cancer is a non-infectious disease caused by uncontrolled cell division or malignant cell growth. Normal cells are still being attacked and destroyed by abnormal cells. Death can occur if these systems are not managed [72]. Therapeutics for many forms of cancer have been developed over the years. However, because normal and cancer cells are so similar, anticancer drugs are likely to have negative effects on normal cells. Furthermore, because anticancer drugs are very toxic, cancer patients usually have side effects after therapeutic procedures [73,74]. These problems explain why research is being conducted to discover new therapeutic agents to replace existing ones. Many metabolites isolated from organisms are being studied for anticancer properties. According to studies, the majority of natural anticancer compounds have no side effects and control the growth of cancer cells [74]. As a result, one of the most significant strategies for cancer control is the quest for effective natural anticancer drugs. The anticancer mechanism of phlorotannins encompasses several pathways that are linked to the development of cancer and the triggering of cell death [75,76,77]. Phlorotannins prevent cancer via increasing cytotoxic T lymphocytes, dendritic cells, the epithelial-mesenchymal transition process, matrix metalloproteinase, phagocyte release, and decreasing SLUG and VEGF expression. Furthermore, phlorotannins modulate apoptosis induction by increasing the expression of apoptosis antigen 1 (APO-1), B-cell lymphoma 2 (Bcl-2) protein, caspase-3, -7, -9, cysteinyl aspartate specific proteinase (casp), and down-regulating the expression of the protein kinase B (AKT) pathway, B-cell lymphoma-extra-large (Bcl-xL) protein, extracellular signal-related kinase (ERK) pathway, FLICE (FADD-like IL-1β-converting enzyme)-inhibitory protein (FLIP), nuclear factor-kB (NF-κB), phosphoinositide 3-kinase (PI3K) pathway, and X-linked inhibitor of apoptosis (XIAP) (Figure 5). Dieckol extracted from *E. cava* was shown to be cytotoxic to A2780 and SKOV3 ovarian cancer cells, as reported by Ahn et al. [78]. Dieckol promoted apoptosis in SKOV3 cells and reduced tumor growth in SKOV3-bearing mouse models without causing severe toxicity. Furthermore, phloroglucinol from *E. cava* inhibited multiplication and triggered apoptosis in MCF-7 human cancer cells [79]. Phloroglucinol induced an increase in apoptosis-related gene expression while decreasing anti-apoptotic gene expression. Another study found that phloroglucinol effectively suppressed breast cancer cells by downregulating SLUG without generating cytotoxicity [80]. Kang et al. [81] discovered that phloroglucinol decreased cell viability and promoted apoptosis in HT-29 colon cancer cells. Phloroglucinol induced apoptosis-related alterations such as Bcl-2 family protein modification, cytochrome c release, and caspase-3 and -8 activation (Figure 5). Phlorotannins isolated from *Cystoseira sedoides* induced apoptosis in more than half of MCF-7 breast cancer cells, with the IC_50_ value of 78 μg/mL [82]. Furthermore, in *N*-nitrosodiethylamine-induced hepatocarcinogenesis, dieckol inhibited lipid peroxidation, hepatic cell damage, and increased antioxidant defense mechanisms (Figure 5) [83].

## 6. Antioxidant Abilities of Phlorotannins in Modulating Oxidative Stress

Oxidation is a key metabolic process in which electrons or hydrogen are transferred from a material to an oxidizing agent. Free radicals are produced during oxidative processes, which damage or destroy cells. Excess-free radical generation destroys enzymes like superoxide, dismutase, catalase, and peroxidase, resulting in a variety of human disorders, including diabetes and cancer [84,85]. The consequences of excessive oxidation highlight the need for a wide range of antioxidants obtained from natural sources. By preventing oxidation, antioxidants serve as a defensive mechanism against the consequences of excessive oxidation. These antioxidants found in the body have been linked to protection from various diseases, including cancer, aging, and Alzheimer’s [51,86]. Various in-vitro methods are applied to determine the antioxidant activity present in natural sources. Scavenging assays utilizing 2,2-diphenyl-1-picrylhydrazyl (DPPH^•^) and 2,2′-azino-bis-3-ethylbenzthiazoline-6-sulphonic acid radical are used to evaluate the radical scavenging activity of antioxidants. The β-carotene-linoleate model systems and the thiobarbituric acid reactive substances tests are widely used to assess the antioxidant activity of foods and organic compounds containing lipids. The reducing power of antioxidants is measured using the ferric antioxidant power reduction and cupric ion reducing antioxidant capacity assays. These assays are commonly used to identify prospective antioxidant sources [87,88]. Sathya et al. [89] found that the phlorotannins from *C. trinodis* had significant radical-scavenging activity against superoxide and DPPH radicals and were as effective as ascorbic acid and α-tocopherol. Previously, it was attempted to determine the relationship between the number of hydroxyl groups in phlorotannins and antioxidant activity. The results showed the antioxidant activity of phlorotannins to be dependent on the structure (substitution pattern) of phlorotannins [90]. Moreover, Ahn et al. [91] demonstrated that phlorotannins isolated from *E. cava* have better free radical scavenging and DNA damage inhibition properties. Nakai et al. [92] conducted a study to enhance the radical scavenging activity of *S. ringgoldianum*. As a result, it was shown that oligomerization of phloroglucinol was required for the improvement of radical scavenging activity. A recent study found a relationship between phlorotannin content and total antioxidant activity, indicating that phlorotannins play a role in determining total antioxidant activity [93]. Lee et al. [94] discovered that phlorofucofuroeckol-A exhibited excellent antioxidant properties against AAPH-induced ROS generation and lipid peroxidation in both in vitro and in vivo systems. Similarly, it was demonstrated that phlorotannins isolated from *E. cava* protected neurons against H_2_O_2_-induced neurotoxicity and significantly reduced intracellular ROS production, lipid peroxidation, and apoptosis generated by AAPH [95,96].

## 7. Phlorotannins with Anti-Adipogenesis, Antidiabetic, and Anti-Obesity Activities

**Figure 6:**
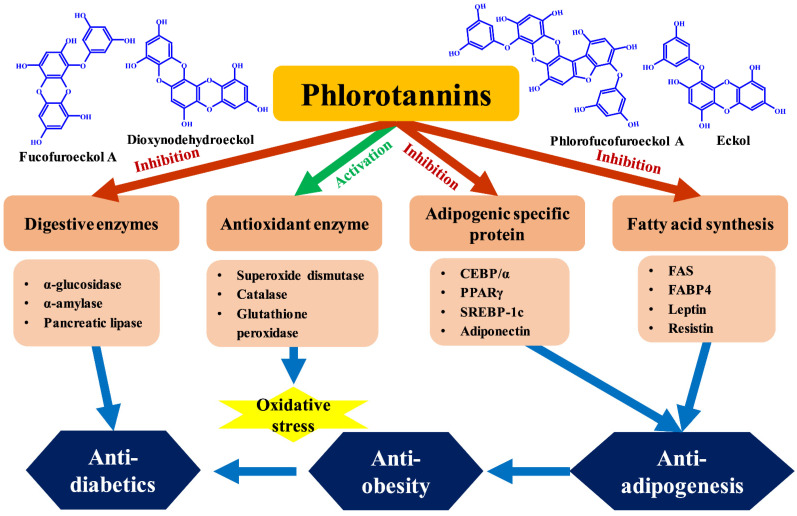
Phlorotannins with anti-adipogenesis, antidiabetic, and anti-obesity activities.

Obesity, diabetes, dyslipidemia, myocardial infarction, stroke, and hypertension are all symptoms of metabolic syndrome [97]. Metabolic syndrome is becoming more common in modern life and is regarded as a major health issue. According to Blüher [98], metabolic syndrome and obesity are closely associated. Obesity is defined by a rise in adipose tissue mass and an increase in fat cell size and number. Adipogenic transcription factors that control enzymes involved in lipid metabolism induce adipogenesis [99]. Transcription factors such as CCAAT/enhancer-binding protein (C/EBPα) and peroxisome proliferator-activated receptor γ (PPARγ), in particular, have a direct influence on adipogenesis [100,101]. Adipocyte hypertrophy and hyperplasia can be induced during adipogenesis by expressing adipogenesis-specific genes [102]. The model utilized to assess adipogenesis and adipocyte differentiation are 3T3-L1 cells [103]. Despite the fact that several anti-obesity drugs have been developed to treat obesity, current anti-obesity treatments have substantial adverse effects. Existing anti-obesity drugs have been linked to major psychological and cardiovascular problems [104]. Given the negative effects of anti-obesity drugs, there is a need for research on natural compounds having anti-obesity efficacy. Hu et al. [105] discovered that phlorotannins had an anti-obesity action by inhibiting pancreatic lipase and protein tyrosine phosphatase 1B and inducing adipocyte differentiation and apoptosis. Dioxynodehydroeckol and fucofuroeckol A isolated from *E. bicyclis* were shown to have substantial antidiabetic action against α-glucosidase (IC_50_ value of 131.34, 93.33 nmol/L) and α-amylase (IC_50_ value of 42.91, 472.7 µmol/L) [106]. Furthermore, fucofuroeckol A and dioxinodehydroeckol inhibited α-glucosidase in a non-competitive manner. Another investigation found that among the *E. cava* phlorotannins, dieckol had the greatest inhibitory effect against α-glucosidase (IC_50_ value of 10.8 µmol/L) and α-amylase (IC_50_ value of 124.9 µmol/L) [107]. Gheda et al. [108] found that providing phlorotannins from *C. compressa* to diabetic rats lowered their serum glucose levels, total serum cholesterol, total triglycerides, liver malondialdehyde, α-amylase, and glucosidase activity. Moreover, the histological investigation revealed that the diabetic group’s islets of Langerhans and certain necrotic regions were substantially damaged. In contrast, sections of diabetic rats treated with phlorotannin exhibited a considerable improvement and recovery in the size of the islets of Langerhans. *Fucus vesiculosus* phlorotannin extract inhibited pancreatic lipase, α-amylase, and notably, µ-glucosidase [109]. Ryu et al. [110] discovered that isophloroglucin A isolated from *Ishige okamurae* had an IC_50_ value of 54.97 μM in α-glucosidase inhibition. It showed the greatest σ-glucosidase inhibitory activity among the phlorotannin groups isolated from *I. okamurae*. The IC_50_ values of phlorofucofuroeckol A for α-glucosidase and α-amylase from *E. cava* were 19.52 and 6.34 μM, respectively [111]. The enzyme activity was inhibited by the hydroxyl group of phlorofucofuroeckol A, which is bound to it. Kang et al. [112] administered dieckol (10 and 20 mg/kg body weight) to diabetic mice for 14 days and observed that the blood sugar levels, serum insulin levels, body weight, and thiobarbituric acid reactive substrates in the dieckol-administered group were significantly decreased compared to the saline-administered group. Furthermore, the dieckol-administered group showed enhanced activity of several antioxidant enzymes e.g., catalase, glutathione peroxidase, and superoxide dismutase. Kim et al. [113] reported that dioxynodehydroeckol reduced lipid accumulation by inhibiting PPARγ, CEBP/α, and sterol regulatory element-binding protein 1 (SREBP). In addition, dioxynodehydroeckol suppressed fatty acid synthase, fatty acid-binding protein, lipoprotein lipase, acyl-CoA synthetase 1, and fatty acid transport protein. In a tyrosinase inhibitor experiment, dieckol from *E. cava* outperformed a commercial tyrosinase inhibitor (kojic acid) [114]. Interestingly, *E. stolonifera* eckol, phlorofucofuroeckol A, and phloroglucinol, inhibited lipid accumulation in 3T3-L1 cells, as well as the expression of key adipocyte marker genes such as C/EBPα and PPARγ [100]. Liu et al. [115] administered dieckol extract (50 mg/kg/day) to nonalcoholic fatty liver induced rats for 4 weeks. Significant improvement was observed in the plasma lipid profile, visceral fat index, liver index, and liver fat accumulation in the dieckol extract administration group. Ko et al. [116] reported that dieckol of *E. cava* exhibited an adipogenesis inhibitory activity that downregulates the expression of SREBP1, PPARγ, and C/EBPα. Figure 6 depicts the diagrammatic explanation of phlorotannin’s activity as an antidiabetic, anti-obesity, and anti-adipogenesis agent via the activation and inhibition of various enzymes and signaling molecules. Table 2 lists a number of other phlorotannins with various biological functions.

## 8. Limitations Related to the Phlorotannin Isolation, Purification, Characterization, and Application

Phlorotannins isolated from seaweed are compounds with high biological activity and have been found to be effective as a natural antibacterial, antiviral, antioxidant, anticancer, and anti-obesity agent. However, several constraints must be overcome in order to take full advantage of these properties. Because certain seaweeds contain heavy metals such as Cu, Hg, As, and Cd, unpolished seaweed can be lethal to humans [117,118,119]. To address this issue, a high level purification technique is required. Although there is a rising interest in bioactive compounds found in seaweed, it is difficult to validate comprehensive in-vivo processes due to a lack of bioavailability research [120,121,122]. Furthermore, in vitro investigations make it difficult to identify a compound’s genuine potential in formulation development [35]. As a result, there is a need for a study to clarify the mechanism of phlorotannins in vivo and clinically. Methanol, ethyl acetate, ethanol, and acetone are the most common solvents used for phlorotannin extraction. These solvents are not safe for animals or humans, and researchers should be aware of the maximum residual limit of solvents in phlorotannins [123,124]. High-temperature extraction of phlorotannins or microwave extraction would dissolve more compounds and increase extraction yield. The heat generated at high temperatures and the high output of microwaves may reduce antioxidants in phlorotannins, hence it is critical to investigate proper extraction conditions [125,126]. Enzyme-assisted extraction of phlorotannins is a safe and ecologically friendly approach. However, due to the cost of enzymes, lack of substrate-specific enzymes, and limitations in maintaining bioreactor conditions, using an appropriate extraction method is required [127,128]. Several experiments have been carried out to identify the structural characteristics of purified phlorotannins, but the drawback is that there is no available library for comparison with the standard [129,130]. The spectrophotometer-based methods, such as the Folin-Ciocâlteu assay, FRAP assay, and ORAC assay, have a low specificity because non-phenolic substances are prone to overestimate the results [131,132]. Although GC-MS technology is capable of detecting pure compounds, its limits come when the analytical technique is more sensitive than the assay employed to evaluate biological activity [133]. This might lead to the loss of trace quantities of biologically active compounds, emphasizing the need to isolate pure molecules. Furthermore, the solvent employed in the extraction should be evaluated critically at each stage of the cycle process. The technique should be clarified fundamentally, including the rationale of the maximum residual limit of solvent within the pure compound. It might be argued that optimizing extraction processes would result in high-quality phlorotannin from various seaweed sources as nutraceuticals for improving human health. However, because most research on the biological use of phlorotannins have used extracts, efforts must be made to develop a standard purification technique for the active component present in the extracts.

**Table 1:** Different types of phlorotannins exhibiting antimicrobial activities and their action mechanisms.

Pure Phlorotannin or Extracts	Sources	Purification Methodology	Microbial Pathogens	Active Concentration	Types of the Testing Method	Action Mechanism	Reference
**Antibacterial**							
8,8′-Bieckol Eckol Dieckol Phloroglucinol Phlorofucofuroeckol A	*Ecklonia kurome*	Silica acid chromatography	MRSA *Escherichia coli* *Bacillus cereus* *Campylobacter jejuni* *Salmonella* Typhimurium *S. enteritidis* *Vibrio parahaemolyticus*	MBC (>6.35 to 0.13 µmol/mL) MBC (>6.35 to 0.27 µmol/mL) MBC (>6.35 to 0.54 µmol/mL) MBC (0.79 to 0.03 µmol/mL) MBC (>6.35 to 0.27 µmol/mL) MBC (>6.35 to 0.27 µmol/mL) MBC (>6.35 to 0.27 µmol/mL)	Broth microdilution method	ND	[21]
Ethyl acetate fraction of methanolic extract Phlorofucofuroeckol Eckol 7-Phloroeckol Dieckol Dioxynodehydroeckol	*Eisenia bicyclis*	Folin-Ciocâlteu method	*Listeria monocytogenes*	MIC (128 to 256 µg/mL) MIC (32 to 128 µg/mL) MIC (128 to 256 µg/mL) MIC (64 to 128 µg/mL) MIC (64 to 128 µg/mL) MIC (64 to 128 µg/mL)	Micro-dilution method	ND	[36]
Crude methanolic extract	*Laminaria digitata* *L. saccharina* *Himanthalia elongata* *Palmaria palmata* *Chondrus crispus* *Enteromorpha spirulina*	Folin-Ciocâlteu method	*L. monocytogenes, S. abony, Enterococcus faecalis*, and *P. aeruginosa*	2.21% to 100% bacterial inhibition	Two-fold dilution method	ND	[37]
Phlorotannins	*Hizikia fusiforme*	ND	*Aeromonas hydrophila* *Chrobacterium violaceum* *E. coli* *Staphylococcus aureus* *P. aeruginosa* *V. parahaemolyticus*	MIC 0.1943 g/mL for *C. violaceum* MIC 0.0972 g/mL for others	Two-fold dilution method	Inhibited QS activity Decreased bacterial motility Inhibited the extracellular protease, pyocyanin, and hemolysin Inhibited biofilm formation	[38]
Ethyl acetate fraction of methanolic extraction Eckol Fucofuroeckol 7-Phloroeckol Dioxynodehydroeckol Dieckol	*E. bicyclis*	ND	MRSA	MIC (32 to 64 µg/mL)	Two-fold dilution method	ND	[39]
Acetone fraction of methanolic extraction Dieckol Phlorofucofuroeckol A	*E. cava*	Sephadex LH-20 column chromatography	*Propinibacterium acnes*	MIC (39 µg/mL)	Broth microdilution assay	ND	[40]
Ethyl acetate fraction of ethanolic extraction Eckol	*E. cava*	Silica gel column chromatography	MRSA *Salmonella* sp.	MIC (125 to 250 µg/mL)	Broth microdilution method	ND	[42]
Ethyl acetate fraction of methanolic extract Phlorotannins	*E. cava*	Reversed-phase column chromatography	*Edwardsiella tarda* *Streptococcus parauberis* *S. iniae* *V. anguillarum* *V. harveyi* *V. scophthalmi*	MIC 128 to 256 µg/mL	Micro broth dilution method	ND	[44]
Eckol Dieckol	*E. arborea*	Column chromatography	*V. parahaemolyticus*	MBC 350 to 5.23 mg/g for eckol MBC 350 to 1.67 mg/g for dieckol	Broth dilution method	Develop antibiotic agents for medicated shrimp feed additive	[45]
Dieckol	*E. stolonifera*	Sephadex LH-20 column chromatography	MRSA	MIC 32 to 64 μg/mL	Two-fold dilution method	ND	[46]
Dieckol Phlorofucofuroeckol-A	*E. bicyclis*	ND	*S. aureus* *S. epidermidis* *P. acnes*	MIC 128 −256 μg/mL	Micro-dilution method	ND	[41]
Phlorotannin	*Sargassum thunbergii*	ND	*V. parahaemolyticus*	900 μg/mL inhibited biofilm formation	Micro-dilution method	ND	[47]
Phlorotannin extraction Phloroglucinol Tetrafucol A Tetraphlortol B Eckol	*Laminaria digitata*	Electron micrograph	Mixed bacterial culture	Biofilm inhibition	Batch assay	ND	[48]
Crude phlorotannins	*Cymbella* spp.	Thin-layer chromatography	*Corynebacterium diphtheriae* *E. coli* *Klebsiella pneumonia* *Proteus mirabilis* *S. aureus* *S.* Typhimurium *P. aeruginosa*	MIC value of 1.56, 1.56, 3.12, >3.12, 3.12, 1.56, 1.56 mg/mL respectively	Micro-dilution method	Inactivated microbial adhesions, enzymes, and cell envelope transport proteins	[134]
**Antifungal**							
Acetone extracted crude extract Phlorotannins	*Cystoseira usneoides* *C. nodicaulis* *Fucus spiralis*	Crude extraction	*Candida albicans* ATCC 10231	MIC 15.6,31.3,31.3 mg/mL for *C. nodicaulis, C. usneoides, and F. spiralis*, respectively	Broth microdilution method	Affected the ergosterol composition of the cell membrane Increased the mitochondrial dehydrogenase Inhibited dimorphic transition of fungi	[22]
Methanolic extraction Dieckol	*E. cava*	Silica-gel chromatography	*Trichophyton rubrum*	MIC 200 µM	Micro broth dilution assay	Changed cytoplasmic integrity	[49]
Ethylacetate fraction of methanolic extract Fucofuroeckol A	*E. bicyclis*	RP-18 open column chromatography and Sephadex LH-20	*C. albicans*	MIC 512 µg/mL	Broth microdilution assay	Induced ROS species Disrupted fungi cell wall	[50]
**Anti-viral**							
Ethylacetate extract of methnol extract Phloroglucinol derivatives 6,6′-Bieckol	*E. cava*	Silica-gel chromatography	HIV-1	EC_50_ 1.72 µM (syncytia production) EC_50_ 1.26 µM (antigen production)	Western blot analysis Cell viability assay	Induced syncytia production Inhibited viral p24 antigen production Inhibited lytic effect	[57]
8,4′-Dieckol	*E. cava*	Silica-gel chromatography	HIV-1	91% Inhibition of reverse transcriptase at 50 µM	Reverse transcriptase assay	Induced syncytia production Inhibited viral p24 antigen production Inhibited lytic effect	[57]
8,4′-Dieckol 8,8′-Bieckol Phlorofucofuroeckol A	*E. cava*	ND	HIV-1	50% Inhibition of reverse transcriptase at 0.51 µM	Reverse transcriptase assay	Inhibited reverse transcriptase enzyme activity	[58]
Ethyl acetate fraction of ethanolic extract Triphloretol Eckol Dioxynodehydroeckol Dieckol 2-Phloroeckol 7-Phloroeckol Phlorofucofuroeckol A Fucodiphloroethol	*E. cava*	Silica-gel chromatography	SARS-CoV 3CL	IC_50_ 2.7 to >200 µM	Cell-free /based analysis SARS-CoV 3CL^pro^ *trans*-cleavage assay	Inhibited 3CL^pro^ hydrolysis	[23]
Phlorofucofuroeckol	*E. cava*	Silica-gel column chromatography	Influenza A virus	IC_50_ 4.5 µM	Chemiluminescent neuraminidase inhibition assay	ND	[59]

**Table 2:** Different types of phlorotannins exhibiting anticancer, anti-inflammatory, antidiabetic, antioxidant, anti-obesity, anti-adipogenesis, and other biological activities.

Pure Phlorotannin or Extracts	Sources	Purification Methodology	Biological Activity	Active Concentration	Types of the Testing Method	Action Mechanism	Reference
**Anticancer**							
6,6′-Bieckol 8,8-Bieckol Dieckol 7-Phloroeckol Phlorofucofuroeckol	*Ecklonia cava*	ND	Ovarian cancer cells undergo apoptosis	IC_50_ 84.3 and 99.6 µg/mL against A2780 and SKOV_3_ cells for ethanolic extract IC_50_ 77.31 and 92.7 µg/mL against A2780 and SKOV_3_ cells for dieckol IC_50_ 77.31 to 137.77 and 96.3 to >200 µg/mL against A2780 and SKOV_3_ cells for others	PI staining MTT assay PI and Annexin double staining Western blot analysis and flow cytometry and SKOV_3_ tumor xenograft model	Inhibited tumor xenograft growth Cytotoxicity effect on ovarian cancer cells (A2780 and SKOV_3_) Caused mitochondria disfunction Suppressed the levels of anti-apoptosis protein Induced ROS Reversed the caspase activation	[78]
Phloroglucinol derivatives Dioxynodehydroeckol 1-(3′,5′-Dihydroxyphenoxy)-7-(2″,4″,6-trihydroxyphenoxy)-2,4,9-trihydroxydibenzo-1,4-dioxin	*E. cava*	Silica-gel column chromatography	Human breast cancer cells	At 100 µM, it inhibited MCF-7 cells growth by 64% Inhibited proliferation of MDA-MB-231 cells 55% at 100 µM	Cell proliferation assay	Anti-proliferative activity on MCF-7 cancer cells Downregulated NF-κB and depended on activated genes Induced caspase activity Induced the cleavage of DNA repair enzyme Induced pro-apoptotic gene Suppressed anti-apoptotic gene	[79]
Phloroglucinol	Brown seaweeds	ND	MDA-MB231 breast cancer cells	IC_50_ 50 µM of migratory and invasive ability of MDA-MB231 breast cancer cells	Western blot analysis Activated RAS affinity precipitation assay Invasion and migration assay	SLUG was suppressed by inhibiting P13K/AKT and RAS/RAF-1/ERK signaling Reduced cancer cell’s metastatic ability	[80]
Phloroglucinol	Seaweeds	ND	Human colon cancer cells HT-29	12.5 µg/mL caused fragmented nuclei and cell shrinkage	Western blot analysis Cell cycle analysis mRNA expression analysis 4′-6′-Diamidino-2-phenylindole staining assay	Downregulated the expression of Ras, Raf, and mitogen-activated protein kinase Downregulated the phosphorylation of the extracellular-signal-regulated kinase Decreased mammalian target of rapamycin and expression of its downstream effectors p70S6 kinase Decreased elF4b and RPS6 translation initiation factor	[135]
Phlorotannins	*Cystoseira sedoides*	Reversed-phase column chromatography	MCF-7 cells (human breast cancer cells)	In MCF-7 cells, the IC_50_ for inducing apoptosis was 78 µg/mL	Double Annexin V-FITC/PI test	Prevented spheroid growth	[82]
Dieckol	*E. cava*	Sephadex LH-20 column chromatography	Protective efficacy against *N*-nitrosdiethylamine -induced rat hepatocarcinogenesis	Alkaline phosphatase, lactate dehydrogenase, transaminase, gamma-glutamyl transferase, total bilirubin, and a-fetoprotein activities increased in NDEA-induced rats given dieckol water (10–40 mg/kg body weight) for 15 weeks	Serum marker enzymes analysis	It prevents hepatic cell damage and lipid peroxidation NDEA-induced hepatocarcinogenesis enhances the enzymatic and non-enzymatic antioxidant defense system	[83]
Isololiolide	*C. tamariscifolia*	Reverse phase preparative HPLC	Anti-proliferative activity	IC_50_ 32.36 µM of cytotoxic activity against gastric cancer cells IC_50_ 23.59 µM of cytotoxic activity against colon cancer cell line IC_50_ 13.15 µM of cytotoxic activity against human hepatocellular carcinoma cells	MTT colorimetric assay	Expression of proteins in the apoptotic cascade Induced apoptosis through the modulation of apoptosis-related proteins.	[136]
**Anti-inflammatory**							
Ethyl acetate fraction of methanolic extraction Phloroglucinol (PG) Phlorofucofuroeckol A (PFA) Eckol (EK) 7-Phloroeckol (7-PE) Dieckol (DE)	*Eisenia bicyclis*	Chromatography on silica gel column	Cell viability and NO production (LPS-induced RAW264.7 cells)	>10 µg/mL cytotoxicity for PG and PFA 24.5% and 66.2% inhibition of NO production for PG and PFA IC_50_ 52.86, 51.42, and 26.87 µg/mL for EK, DE, 7-PE	MTT assay	Inhibited NF-κB -related inflammatory gene expression via ROS inhibition Inhibited LPS-induced nitric oxide and butyl hydroperoxide-induced ROS production	[64]
Phloroglucinol	*E. cava*	ND	Inhibition of oxidative stress and inflammation	Inhibition of protein oxidation (90% at 10 µM) Inhibition of TNF-α, IL-1β (30% at 10 µM) Inhibition of PGE_2_ (40% at 10 µM)	MTT assay Membrane protein oxidation assay Enzyme immunoassay Western blot analysis	Inhibited oxidative stress Inhibited the production of IL-6, TNF-α, IL-1β, and PGE_2_ Matrix metalloproteinase expression was reduced	[66]
Phlorotannins	*Bifurcaria bifurcata*	Reversed-phase column chromatography	Cytotoxic effect on ATDC-5 mouse model cell lines	50% inhibition of cell growth in ATDC-5 cells at 100 µM	MTT assay	ND	[68]
Phlorotannins	*Padina tetrastromatica*	Reversed-phase column chromatography	Effect on THP-1 cell viability	From 1.5 to 50.0 µg/mL	MTT assay	Anti-MRSA potential Enhances the high-glucose-induced pro-MMP-9 expression	[69]
Phloroglucinol	Brown algae	ND	Anti-inflammatory effect and oxidative stress on RAW264.7 and HT1080 cells	From 1 to 100 µM	MTT assay	Inhibitory effects on oxidative stress and the production of inflammatory mediators such as IL-6, TNF-a, IL-1β, and PGE2 in RAW264.7 cells stimulated by LPS Decreased the expression of matrix metalloproteinase in HT1080 cells	[66]
Phlorotannins	*Fucus guiry* *F. serratus* *F. sprialis* *F. vesiculosus*-wild *F. vesiculous*-aquaculture	Aqueous acetone extraction	Anti-inflammatory and toxicity capability in RAW 264.7 macrophages and cell-free systems	IC_50_ 82.10, 110.16, 362.42, 364.84, > 500 μg DE/mL for LOX inhibition, respectively IC_50_ 451.91, 1214.73, 801.97, 1330.61, 2072.32 μg DE/mL for NO scavenging, respectively IC_25_ 97.73, 77.04, 95.86, 56.52, 317.41 μg DE/mL for NO reduction, respectively Nontoxic at 31.25–500 μg/mL in RAW 264.7 macrophages stimulated with bacterial LPS	MTT assay	Function in inflammatory conditions, acting on both enzymatic and non-enzymatic inflammatory targets	[65]
Phlorofucofuroeckol B (PFF-B)	*E. stolonifera*	High-performance chromatography	PFF-B inhibits the generation of inflammatory mediators induced by LPS	Decreased secretion of pro-inflammatory cytokines, including TNF-α, IL-1β, and IL-6 Decreased expression of pro-inflammatory proteins such as COX-2 and inducible NO synthase	MTS assay NF-κB promoter/luciferase assay	By limiting the breakdown of the inhibitor κb-α, the activation of nuclear factor kappaB was prevented. Inhibited the phosphorylation of Akt, ERK, and JNK	[70]
6,6′-Bieckol	*E. cava*	ND	LPS-stimulated macrophage RAW 264.7 cells have anti-inflammatory effects	Inhibited NO and PGE_2_ production at concentrations of 100, 200 μM Suppressed the LPS-induced expression of COX-2 and inducible-NOS and at the mRNA and protein levels TNF-α and IL-6 mRNA expression were reduced	MTT assay PGE_2_ assay Chromatin immunoprecipitation assay	Downregulation of COX-2, iNOS, and pro-inflammatory cytokines in LPS-stimulated macrophages via the NF-κB pathway	[71]
Phlorofucofuroeckol B	*Myagropsis myagroides*	High-performance chromatography	Anti-inflammatory activity	Inhibited LPS-induced PGE_2_ and NO production Inhibited COX-2 and iNOS Pro-inflammatory cytokines were inhibited, as well as NF-κB activation and translocation	MTS assay Transient transfection and luciferase assay	In LPS-stimulated macrophage cells, the NF-κB pathway was inhibited by inhibiting the phosphorylation of ERKs and Akt	[67]
**Antidiabetic**							
Ethyl acetate fraction of methanolic extraction Dioxynodehydroeckol (DDE) Fucofuroeckol (FFE)	*E. bicyclis*	Chromatography and nuclear magnetic resonance	Antidiabetic activity	IC_50_ 131.34 nmol/L for α-glucosidase and 42.91 µmol/L for α-amylase for FFE IC_50_ 93.33 nmol/L for α-glucosidase and 472.7 µmol/L for α-amylase for DDE	Enzymatic inhibitory assay	Inhibition of α-amylase and α-glucosidase enzyme activities	[106]
Phlorofucofuroeckol Dieckol 6-6′-Bieckol 7-Phloroeckol Fucodiphloroetho	*E. cava*	Column chromatography using silica gel	antidiabetic activity	IC_50_ 10.75 to 49.49 µmol/mL for α-glucosidase and >500 to 124.98 µmol/L for α-amylase	Enzymatic inhibitory assay	Inhibition of α-amylase and α-glucosidase enzymes activities	[107]
Phlorotannins	*C. compressa*	UPLC-MS/MS	Antidiabetic activity	After four weeks of diabetes induction, diabetics were treated with 60 mg/kg of phlorotannin extract.	MTT assay	Reduced serum glucose, malondialdehyde, glucosidase, and α-amylase activity in the liver Reduced damage in pancreatic β cells	[108]
Ethylacetate fraction of acetone extract	*F. vesiculosus*	Mass spectroscopy (UHPLC-MS)	Antidiabetic and anti-obesity activity	IC_50_ for α-amylase 2.8 µg/mL IC_50_ for α-glucosidase 0.82 µg/mL IC_50_ for pancreatic lipase 0.82 µg/mL	α-amylase, α-glucosidase, pancreatic lipase inhibitory assay	Inhibited α-amylase, α-glucosidase, and pancreatic lipase enzymes Delayed carbohydrates and lipid digestion	[109]
Ishophloroglucin A	*Ishige okamurae*	Semipreparative HPLC column	Anti-α-glucosidase activity	IC_50_ value of 54.97 µM in α-glucosidase inhibition	α-Glucosidase inhibitory assay	Inhibited α-glucosidase	[110]
Phlorofucofuroeckol A	*E. cava*	Electrospray ionization-multistage tandem mass spectrometry and HPLC	Antidiabetic activity	IC_50_ for α-amylase 6.34 µM IC_50_ for α-glucosidase 19.52 µM	α-Glucosidase inhibitory assay α-Amylase inhibitory assay	Inhibition of α-amylase and α-glucosidase enzymes activities	[111]
Dieckol	*E. cava*	Reversed-phase HPLC	Antidiabetic activity	Dieckol administration reduced blood glucose levels, serum insulin levels, and body weight Increased activity of anti-oxidant enzymes in liver tissues, including superoxide dismutase, catalase, and glutathione peroxidase	Serum glucose content reduced Lipid peroxidation production Catalase and superoxide dismutase activities	Activates both the AMPK and PKB signaling cascades	[112]
**Antioxidant**							
Dichloromethane fraction of methanolic extraction	*C. trinodis* (Forsskal) *C. Agardh*	Silica-gel column chromatography	Antioxidant activity	69.62% radical scavenging activity	DPPH radical scavenging	ND	[89]
Dichloromethane fraction of ethanolic extraction 974-A 974B Phloroglucinol Dieckol	*E. curome*	Sequential chromatography on two reverse phase column	Antioxidant activity	IC_50_ 10, 11, 110, 10 µM respectively	DPPH radical scavenging	Reduced intracellular reactive oxygen species	[90]
Ethylacetate fraction of methanolic extract Phloroglucinol Eckol Dieckol	*E. cava*	Sephadex LH-20 column chromatography	Antioxidant activity	90% radical scavenging activity for eckol at 0.25 to 1 mg/mL 100% hydroxyl radical scavenging activity for dieckol at 0.25 mg/mL 90% alkyl radical scavenging activity for eckol at 0.05 mg/mL 68.96% to 50% DNA damage values for all three at 25 µg/mL	DPPH radical assay Hydroxyl radical assay Alkyl radical assay Superoxide radical assay Comet assay (Protecting effects against H_2_O_2_-mediate DNA damage	Inhibited free radical activities Damaged DNA	[91]
Ethanolic extract Phlorotannins	*Sargassum inggoldianm*	Matrix-assisted laser desorption ionization time-of-flight mass spectroscopy	Antioxidant activity	IC_50_ 1.0 µg/mL	Electron spin resonance spectrometry	Showed superoxide anion radical scavenging activity	[92]
Phlorotannins	*S. dupplicatum*	Sephadex LH-20 column chromatography	Antioxidant activity	Total antioxidant activity 11.17 ± 0.28 mg ascorbic acid equivalent/g DW Reducing power activity by 11.09 ± 0.24 mg FeSO_4_ equivalent/g DW	Antioxidant activity Reducing power activity	ND	[93]
Phlorofucofuroeckol-A	*E. cava*	Centrifugal partition chromatography	Antioxidant activity	Scavenging activity against alkyl radicals, with an IC_50_ value of 3.9 µM With an IC_50_ value of 21.4 µM, it has a scavenging action against hydroxyl radicals With an IC_50_ value of 10.3 µM, it has a scavenging action against DPPH radicals	Assay for alkyl radical scavenging capacity Assay for hydroxyl radical scavenging capacity DPPH assay	Antioxidant and lipid peroxidation protection Malondialdehyde inhibition in AAPH-stimulated Vero cells	[94]
Dieckol Eckol Eckstolonol Phloroglucinol Triphloroethol A	*E. cava*	HPLC	Neuroprotective against H_2_O_2_-induced cellular damage in HT22 neuronal cells from the murine hippocampus	ROS levels were reduced to 75.22%, 69%, 67.32%, 77.63%, 77.73%, respectively (treatment of H_2_O_2_-treated cells at a concentration of 50 M) The apoptotic sub-G1 DNA content was reduced to 9.55%, 13.03%, 5.55%, 6.61%, 3.03%, respectively (pre-treatment with 50 M phlorotannins)	Scavenging efficacy on ROS production Neuroprotective effects in H_2_O_2_-treated HT22 cells	Remove intracellular ROS and inhibit ROS accumulation Inhibited H_2_O_2_-induced Ca^2+^ release	[96]
Dieckol Eckol Eckstolonol Phloroglucinol Triphloroethol A	*E. cava*	Sephadex LH-20 column chromatography	The ability of phlorotannins to scavenge ROS in AAPH-induced zebrafish embryos	Reduced intracellular ROS buildup to DCF-DA intensity of 1568, 2346, 1703, 1540, and 2262, respectively (50 µM phlorotannins + 25 mM AAPH)	ROS generation in AAPH-induced zebrafish embryos	Antioxidant efficacy against AAPH-mediated toxicity	[95]
**Anti-obesity**							
Ethylacetate fraction of methanol extraction 6-6′-Bieckol Dieckol Phlorofucofuroeckol A	*E. cava*	Sephadex LH-20 column chromatography	Inhibition of adipogenesis	Inhibited lipid accumulation 60%, 40% 20% at 100 µL respectively	Oil-Red O staining method	Downregulated adipogenic specific genes (SREBP-1, C/EBPα, FABP4, and PPARγ)	[116]
Ethylacetate fraction of ethanolic extraction Dioxynodehydroeckol	*E. cava*	Silica-gel column chromatography	Inhibition of adipogenesis	Inhibited adipogenesis 20 µM	Oil-Red O staining method	Reduced lipid accumulation Downregulated the expression of adipogenic specific genes of 3T3-L1 (C/EBPα, SREBP1, and PPARγ) Activated and modulated AMPK signaling pathway	[113]
Diethyl ether fraction of methanolic extract Dieckol Eckol	*E. cava*	Sephadex LH-20 column chromatography	Inhibition of melanogenesis (UV-B radiation-induced cell damage protection effect)	92.7% (dieckol) and 62.4% (eckol) inhibitory effect on tyrosinase at 100 µM	Tyrosinase inhibition assay DCFH-DA, MTT, comet assay	Inhibited tyrosinase Inhibited melamine syntheses	[114]
**Anti-adipogenesis**							
Ethylacetate fraction of methanolic extraction Phloroglucinol Eckol Dieckol Dioxynodehydroeckol Phlorofucofuroeckol A	*E. stolonifera*	Sephadex LH-20 column chromatography	Inhibition of adipogenesis	Inhibited adipogenesis (12.5–100 µM)	Oil Red O staining	Downregulated adipogenic specific genes of 3T3-L1 (C/EBPα and PPARγ)	[100]
Dieckol	*Laminaria japonica*	Silica gel resin absorption	Anti-fatty liver activity	Body weight gain, plasma lipid profiles, visceral fat index, liver index, and hepatic fat deposition were improved in high-fat diet mice given a dieckol-enriched extract (50 mg/kg/day) for four weeks	Histopathological analysis	Advantageous effects on hepatic lipid metabolism Stimulation of hepatic fatty acid β-oxidation	[115]
Dieckol	*E. cava*	Reverse-phase high-performance liquid chromatography	Inhibitory effect on adipogenesis	Inhibition of adipogenesis Peroxisome proliferator-activated receptor, CCAAT/enhancer-binding proteins, fatty acid-binding protein, and SREBP1 expression were all reduced	MTT assay	Activated AMP-activated protein kinase	[116]
**Other biological activities**							
Ethyl acetate fraction of methanolic extract Phloroglucinol	*E. cava*	Sephadex LH-20 column chromatography	Cytoprotective effect against oxidative stress-induced cell damage in (V79-4) Chinese hamster lung fibroblast	65% DPPH radical scavenging activity at 10 µg/mL 70% H_2_O_2_ scavenging activity at 10 µg/mL 26% hydroxy radicals scavenging activity at 10 µg/mL 73% Intracellular reactive oxygen scavenging activity at 10 µg/mL	Radical scavenging activity assay MTT assay Western blot analysis	Scavenge 1,1-diphenyl-2-picrylhydrazyl (DPPH) radical Scavenge H_2_O_2_, hydroxy radicals Prevented lipid peroxidation Reduced intracellular reactive oxygen species Increased the activity of catalase Increased phosphorylation of the extracellular signal-regulated kinase	[137]
Ethyl acetate fraction of methanolic extract Phloroglucinol	*E. cava*	Sephadex LH-20 column chromatography	Radioprotective effect of cells against γ-ray radiation-induced oxidative damage	Phloroglucinol-treated cells reduced irradiation DNA damage by 27% Cell survival rose to 66% in irradiated cells at 10 µg/mL phloroglucinol	Laser scanning microscopy Colorimetric assay Lipid peroxidation assay Western blot analysis Comet assay	Reduced the amount of radiation-induced intracellular ROS Reduced cellular component (lipid, DNA, and protein) damage Reduced the radiation-induced loss of mitochondrial membrane action potential Reduced the active levels of caspase-3 and 9	[138]
Phloroglucinol	Seaweeds	ND	UVB-induced photoaging of human HaCaT keratinocytes	10 µg/mL protected HaCaT keratinocytes against UVB-induced cytotoxicity 10 µg/mL inhibited the accumulation of UVB-induced MMP-1 mRNA and protein at 48 h	UV/visible light absorption analysis Intracellular ROS measurement Western blot analysis Human active MMP-1 Fluorokine E fluorescent assay Chromatin immunoprecipitation assay	Upregulated MMP-1 mRNA and protein activity Augmented intracellular Ca^2+^ level Phosphorylation of mitogen-activated protein kinases Enhanced the activator protein-1 (AP-1) binding to the MMP-1 promotor	[139]
Phlorotannins	*E. radiata*	High-performance counter-current chromatography	Neuroprotective activity	In neuronal PC-12 cells, it was nontoxic up to 50 µM Cholinesterase inhibitory activity at IC_50_ value of 41 µM	MTT assay Acetylcholinesterase inhibitory assay	Elicited neuroprotective activity against amyloid β protein, Aβ1–42 Inhibited Aβ1–42 aggregation, AChE activity, and ROS formation in PC-12 cells Neuroprotective effects through multiple pathways	[140]
Phlorotannins	*E. cava*	Sephadex LH-20 column chromatography	MG63 cell survival and calcium deposition on polycaprolactone (PCL/Ph) micro nanofibres	Increased cell survivability on PCL/Ph micro nanofibers as phlorotannin content increased Increased calcium mineralization on the PCL/Ph micro nanofibers after 14 days	MTT assay	Enhanced bone tissue growth	[116]
Dioxynodehydroeckol	*E. cava*	Silica-gel column chromatography	UVB-induced apoptosis prevention in human keratinocyte (HaCaT) cells	Reduced by 1.83% at 20 µM of DHE compared to 13.31% in cells exposed to UVB	Flowcytometry following Annexin V and PI labeling	ND	[141]
Dieckol	*E. cava*	Sephadex LH-20 with MeOH	Anti-proliferative and anti-angiogenic effect on EA. hy926 cell lines induced with vascular endothelial growth factor	Nontoxic up to 100 µM in EA. hy926 cells Inhibition activity of AP-N enzyme with an IC_50_ value of 52.80 µM Dieckol at the concentration of 10, 50, and 100 µM inhibited vascular endothelial growth factor-induced cell proliferation.	MTT assay Aminopeptidase-N enzyme assay Cell proliferation assay	Cell migration was inhibited by lowering the level of protein and gene expression of matrix metalloproteinases such as MMP-2 and -9	[142]

## 9. Conclusions and Future Perspectives

In conclusion, the present review paper describes the many forms of phlorotannins produced from various marine algae sources. These phlorotannins, which are polyphenolic compounds derived from brown algae, have a wide range of biological characteristics, including antimicrobial, antidiabetic, anticancer, and anti-inflammatory properties (Table 1 and Table 2). The vast spectrum of biological activities associated with phlorotannins is projected to increase their favorable health value in the food, pharmaceutical, and cosmeceutical industries. Despite the prospective uses of phlorotannins, the successful development of a series of products produced from brown algal polyphenols as nutraceuticals have remained an unmet goal. Nutritional epidemiology studies demonstrate a strong link between consuming polyphenols and modifying the molecular pathways of carcinogenesis, as well as lowering cancer cell growth. Despite its extensive use as an antimicrobial, anticancer, antidiabetic, and anti-inflammatory agent, phlorotannin’s low stability and solubility restrict its application. In recent trends, nanotechnology, particularly nanocarriers and nanoformulation, are evolving to overcome the limits associated with natural products and allow targeted and controlled drug delivery. To treat microbial infections, the phlorotannin must be loaded onto the nanocarrier (metallic or nonmetallic) in such a way that the nanocarriers are biocompatible and have inherent antimicrobial capabilities allowing for the synergistic killing of the microbial pathogens [143].

Furthermore, combination therapy has emerged as a viable strategy for revitalizing antibiotics or antifungals in order to fight microbial pathogens in a synergistic manner. The reported chemical structures for some of the phlorotannins (Figure 1) can be employed as scaffolds for the synthesis of the derivatives by grafting some of the active molecules on the phlorotannin to produce broad-spectrum and effective biological effects. Studying the structure-activity relationships of phlorotannins is essential in order to explain the molecular action mechanisms in various biological roles, since various antimicrobial resistance signaling proteins and transcription factors, such as efflux pumps, biofilms, virulence, reverse transcriptase, protease, integrase, and so on, have been thoroughly described in microbial pathogens. As a result, elucidating the molecular docking-interaction of phlorotannins with known microbial proteins relevant to pathogenesis and resistance mechanisms will be more intriguing in future studies. Aside from gene expression studies of microbial virulence, biofilms, antimicrobial resistance genes, and genes implicated in cancer signaling pathways, inflammation, diabetes, and obesity would provide more supporting research to validate the results of the in vitro studies. In addition, to confirm the in vitro antimicrobial activity of phlorotannins, an in vivo investigation employing an animal model organism is required.

## References

[1] Ghosh S., Sarkar T., Pati S., Kari Z.A., Edinur H.A., Chakraborty R. (2022). Novel Bioactive Compounds from Marine Sources as a Tool for Functional Food Development. Front. Mar. Sci..

[2] Bamunuarachchi N.I., Khan F., Kim Y.M. (2021). Antimicrobial Properties of Actively Purified Secondary Metabolites Isolated from Different Marine Organisms. Curr. Pharm. Biotechnol..

[3] Lomartire S., Gonçalves A.M.M. (2022). An Overview of Potential Seaweed-Derived Bioactive Compounds for Pharmaceutical Applications. Marine Drugs.

[4] Singh I.P., Sidana J., Domínguez H. (2013). Chapter 5—Phlorotannins. Functional Ingredients from Algae for Foods and Nutraceuticals.

[5] Yang H., Zeng M., Dong S., Liu Z., Li R. (2010). Anti-proliferative activity of phlorotannin extracts from brown algae *Laminaria japonica* Aresch. Chin. J. Oceanol. Limnol..

[6] Isaza Martínez J.H., Torres Castañeda H.G. (2013). Preparation and Chromatographic Analysis of Phlorotannins. J. Chromatogr. Sci..

[7] Yang B., Fang D., Lv Q., Wang Z., Liu Y. (2021). Targeted Therapeutic Strategies in the Battle Against Pathogenic Bacteria. Front. Pharmacol..

[8] Spitzer M., Robbins N., Wright G.D. (2017). Combinatorial strategies for combating invasive fungal infections. Virulence.

[9] Meganck R.M., Baric R.S. (2021). Developing therapeutic approaches for twenty-first-century emerging infectious viral diseases. Nat. Med..

[10] Ahmad B., Serpell C.J., Fong I.L., Wong E.H. (2020). Molecular Mechanisms of Adipogenesis: The Anti-adipogenic Role of AMP-Activated Protein Kinase. FFront. Mol. Biosci..

[11] Hurt R.T., Kulisek C., Buchanan L.A., McClave S.A. (2010). The obesity epidemic: Challenges, health initiatives, and implications for gastroenterologists. Gastroenterol. Hepatol..

[12] Chakraborty S., Rahman T. (2012). The difficulties in cancer treatment. Ecancermedicalscience.

[13] Yang X., Yang H., Zhou G., Zhao G.-P. (2008). Infectious Disease in the Genomic Era. Annu. Rev. Genom. Hum. Genet..

[14] Reedy J.L., Bastidas R.J., Heitman J. (2007). The Virulence of Human Pathogenic Fungi: Notes from the South of France. Cell Host Microbe.

[15] Heise M.T. (2014). Viral Pathogenesis. Reference Module in Biomedical Sciences.

[16] Peterson E., Kaur P. (2018). Antibiotic Resistance Mechanisms in Bacteria: Relationships Between Resistance Determinants of Antibiotic Producers, Environmental Bacteria, and Clinical Pathogens. Front. Microbiol..

[17] Dhingra S., Rahman N.A.A., Peile E., Rahman M., Sartelli M., Hassali M.A., Islam T., Islam S., Haque M. (2020). Microbial Resistance Movements: An Overview of Global Public Health Threats Posed by Antimicrobial Resistance, and How Best to Counter. Front. Public Health.

[18] van Duin D., Paterson D.L. (2016). Multidrug-Resistant Bacteria in the Community: Trends and Lessons Learned. Infect. Dis. Clin N. Am..

[19] Arendrup M.C., Patterson T.F. (2017). Multidrug-Resistant Candida: Epidemiology, Molecular Mechanisms, and Treatment. J. Infect. Dis..

[20] Villa T.G., Feijoo-Siota L., Rama J.L.R., Ageitos J.M. (2017). Antivirals against animal viruses. Biochem. Pharmacol..

[21] Nagayama K., Iwamura Y., Shibata T., Hirayama I., Nakamura T. (2002). Bactericidal activity of phlorotannins from the brown alga *Ecklonia kurome*. J. Antimicrob. Chemother..

[22] Lopes G., Pinto E., Andrade P.B., Valentão P. (2013). Antifungal activity of phlorotannins against dermatophytes and yeasts: Approaches to the mechanism of action and influence on *Candida albicans* virulence factor. PLoS ONE.

[23] Park J.-Y., Kim J.H., Kwon J.M., Kwon H.-J., Jeong H.J., Kim Y.M., Kim D., Lee W.S., Ryu Y.B. (2013). Dieckol, a SARS-CoV 3CL(pro) inhibitor, isolated from the edible brown algae *Ecklonia cava*. Bioorganic Med. Chem..

[24] Klil-Drori A.J., Azoulay L., Pollak M.N. (2017). Cancer, obesity, diabetes, and antidiabetic drugs: Is the fog clearing?. Nat. Rev. Clin. Oncol..

[25] Scully T., Ettela A., LeRoith D., Gallagher E.J. (2021). Obesity, Type 2 Diabetes, and Cancer Risk. Front. Oncol..

[26] Zatterale F., Longo M., Naderi J., Raciti G.A., Desiderio A., Miele C., Beguinot F. (2020). Chronic Adipose Tissue Inflammation Linking Obesity to Insulin Resistance and Type 2 Diabetes. Front. Physiol..

[27] Ediriweera M.K., Tennekoon K.H., Samarakoon S.R. (2017). A review on ethnopharmacological applications, pharmacological activities, and bioactive compounds of *Mangifera indica* (Mango). Evid.-Based Complementary Altern. Med..

[28] Boucher H.W., Talbot G.H., Bradley J.S., Edwards J.E., Gilbert D., Rice L.B., Scheld M., Spellberg B., Bartlett J. (2009). Bad bugs, no drugs: No ESKAPE! An update from the Infectious Diseases Society of America. Clin. Infect. Dis..

[29] Zacchino S.A., Butassi E., Di Liberto M., Raimondi M., Postigo A., Sortino M.J.P. (2017). Plant phenolics and terpenoids as adjuvants of antibacterial and antifungal drugs. Phytomedicine.

[30] Da Silva C.M., da Silva D.L., Modolo L.V., Alves R.B., de Resende M.A., Martins C.V., de Fátima Â.J. (2011). Schiff bases: A short review of their antimicrobial activities. J. Adv. Res..

[31] Li T., Li L., Du F., Sun L., Shi J., Long M., Chen Z.J.M. (2021). Activity and Mechanism of Action of Antifungal Peptides from Microorganisms: A Review. Molecules.

[32] Dedeurwaerdere S., Friedman A., Fabene P.F., Mazarati A., Murashima Y.L., Vezzani A., Baram T.Z. (2012). Finding a better drug for epilepsy: Antiinflammatory targets. Epilepsia.

[33] Shannon E., Abu-Ghannam N. (2016). Antibacterial derivatives of marine algae: An overview of pharmacological mechanisms and applications. Mar. Drugs.

[34] Shrestha S., Zhang W., Smid S.D. (2021). Phlorotannins: A review on biosynthesis, chemistry and bioactivity. Food Biosci..

[35] Besednova N.N., Andryukov B.G., Zaporozhets T.S., Kryzhanovsky S.P., Kuznetsova T.A., Fedyanina L.N., Makarenkova I.D., Zvyagintseva T.N. (2020). Algae polyphenolic compounds and modern antibacterial strategies: Current achievements and immediate prospects. Biomedicines.

[36] Kim H.-J., Dasagrandhi C., Kim S.-H., Kim B.-G., Eom S.-H., Kim Y.-M. (2018). In vitro Antibacterial Activity of Phlorotannins from Edible Brown Algae, *Eisenia bicyclis* Against Streptomycin-Resistant *Listeria monocytogenes*. Indian J. Microbiol..

[37] Cox S., Abu-Ghannam N., Gupta S. (2010). An assessment of the antioxidant and antimicrobial activity of six species of edible Irish seaweeds. Int. Food Res. J..

[38] Tang J., Wang W., Chu W. (2020). Antimicrobial and Anti-Quorum Sensing Activities of Phlorotannins From Seaweed (*Hizikia fusiforme*). Front. Cell. Infect. Microbiol..

[39] Eom S.-H., Kim D.-H., Lee S.-H., Yoon N.-Y., Kim J.H., Kim T.H., Chung Y.-H., Kim S.-B., Kim Y.-M., Kim H.-W. (2013). In vitro Antibacterial Activity and Synergistic Antibiotic Effects of Phlorotannins Isolated from *Eisenia bicyclis* Against Methicillin-Resistant *Staphylococcus aureus*. Phytother. Res..

[40] Choi J.-S., Lee K., Lee B.-B., Kim Y.-C., Kim Y.D., Hong Y.-K., Cho K.K., Choi I.S. (2014). Antibacterial activity of the phlorotannins dieckol and phlorofucofuroeckol-A from *Ecklonia cava* against *Propionibacterium acnes*. Bot. Sci..

[41] Lee J.-H., Eom S.-H., Lee E.-H., Jung Y.-J., Kim H.-J., Jo M.-R., Son K.-T., Lee H.-J., Kim J.H., Lee M.-S. (2014). In vitro antibacterial and synergistic effect of phlorotannins isolated from edible brown seaweed *Eisenia bicyclis* against acne-related bacteria. Algae.

[42] Choi J.G., Kang O.H., Brice O.O., Lee Y.S., Chae H.S., Oh Y.C., Sohn D.H., Park H., Choi H.G., Kim S.G. (2010). Antibacterial activity of *Ecklonia cava* against methicillin-resistant *Staphylococcus aureus* and *Salmonella* spp.. Foodborne Pathog. Dis..

[43] Mittal N., Tesfu H.H., Hogan A.M., Cardona S.T., Sorensen J.L. (2019). Synthesis and antibiotic activity of novel acylated phloroglucinol compounds against methicillin-resistant *Staphylococcus aureus*. J. Antibiot..

[44] Kim J.-H., Kim S.-B., Hwang H.-J., Kim Y.-M., Lee M.-S. (2016). Antibacterial Property of *Ecklonia cava* Extract against Marine Bacterial Pathogens. J. Food Hyg. Saf..

[45] González-Colunga D., Antunes-Ricardo M., Gutiérrez-Uribe J.A., Cruz-Suárez L.E. (2019). Bioactivity-guided identification of anti-AHPND (acute hepatopancreatic necrosis disease) metabolites of *Ecklonia arborea*. J. Appl. Phycol..

[46] Lee D.-S., Kang M.-S., Hwang H.-J., Eom S.-H., Yang J.-Y., Lee M.-S., Lee W.-J., Jeon Y.-J., Choi J.-S., Kim Y.-M. (2008). Synergistic effect between dieckol from *Ecklonia stolonifera* and β-lactams against methicillin-resistant *Staphylococcus aureus*. Biotechnol. Bioprocess Eng..

[47] Wei Y., Liu Q., Xu C., Yu J., Zhao L., Guo Q. (2016). Damage to the Membrane Permeability and Cell Death of *Vibrio parahaemolyticus* Caused by Phlorotannins with Low Molecular Weight from *Sargassum thunbergii*. J. Aquat. Food Prod. Technol..

[48] Hierholtzer A., Chatellard L., Kierans M., Akunna J.C., Collier P.J. (2013). The impact and mode of action of phenolic compounds extracted from brown seaweed on mixed anaerobic microbial cultures. J. Appl. Microbiol..

[49] Lee M.H., Lee K.B., Oh S.M., Lee B.H., Chee H.Y. (2010). Antifungal activities of dieckol isolated from the marine brown alga *Ecklonia cava* against *Trichophyton rubrum*. J. Korean Soc. Appl. Biol. Chem..

[50] Kim K.-H., Yu D., Eom S.-H., Kim H.-J., Kim D.-H., Song H.-S., Kim D.-M., Kim Y.-M. (2018). Fucofuroeckol-A from edible marine alga *Eisenia bicyclis* to restore antifungal activity of fluconazole against fluconazole-resistant *Candida albicans*. J. Appl. Phycol..

[51] Balakrishnan D., Kandasamy D., Nithyanand P. (2014). A review on antioxidant activity of marine organisms. Int. J. Chem. Technol. Res..

[52] Ma L., Yao L.J.M. (2020). Antiviral effects of plant-derived essential oils and their components: An updated review. Molecules.

[53] Kausar S., Said Khan F., Ishaq Mujeeb Ur Rehman M., Akram M., Riaz M., Rasool G., Hamid Khan A., Saleem I., Shamim S., Malik A. (2021). A review: Mechanism of action of antiviral drugs. Int. J. Immunopathol. Pharmacol..

[54] Riccio G., Ruocco N., Mutalipassi M., Costantini M., Zupo V., Coppola D., de Pascale D., Lauritano C.J.B. (2020). Ten-year research update review: Antiviral activities from marine organisms. Biomolecules.

[55] Besednova N.N., Andryukov B.G., Zaporozhets T.S., Kryzhanovsky S.P., Fedyanina L.N., Kuznetsova T.A., Zvyagintseva T.N., Shchelkanov M.Y. (2021). Antiviral effects of polyphenols from marine algae. Biomolecules.

[56] Sansone C., Brunet C., Noonan D.M., Albini A.J.A. (2020). Marine algal antioxidants as potential vectors for controlling viral diseases. Antioxidants.

[57] Karadeniz F., Kang K.-H., Park J.W., Park S.-J., Kim S.-K. (2014). Anti-HIV-1 activity of phlorotannin derivative 8,4‴-dieckol from Korean brown alga *Ecklonia cava*. Biosci. Biotechnol. Biochem..

[58] Ahn M.J., Yoon K.D., Min S.Y., Lee J.S., Kim J.H., Kim T.G., Kim S.H., Kim N.G., Huh H., Kim J. (2004). Inhibition of HIV-1 reverse transcriptase and protease by phlorotannins from the brown alga *Ecklonia cava*. Biol. Pharm. Bull..

[59] Ryu Y.B., Jeong H.J., Yoon S.Y., Park J.Y., Kim Y.M., Park S.J., Rho M.C., Kim S.J., Lee W.S. (2011). Influenza virus neuraminidase inhibitory activity of phlorotannins from the edible brown alga *Ecklonia cava*. J. Agric. Food Chem..

[60] Afroz M., Zihad S.N.K., Uddin S.J., Rouf R., Rahman M.S., Islam M.T., Khan I.N., Ali E.S., Aziz S., Shilpi J.A. (2019). A systematic review on antioxidant and antiinflammatory activity of Sesame (*Sesamum indicum* L.) oil and further confirmation of antiinflammatory activity by chemical profiling and molecular docking. Phytother. Res..

[61] Beg S., Swain S., Hasan H., Barkat M.A., Hussain M.S.J.P.R. (2011). Systematic review of herbals as potential anti-inflammatory agents: Recent advances, current clinical status and future perspectives. Pharmacogn. Rev..

[62] Patel S.S., Savjani J.K. (2015). Systematic review of plant steroids as potential antiinflammatory agents: Current status and future perspectives. J. Phytopharm..

[63] Barbosa M., Lopes G., Andrade P.B., Valentão P. (2019). Technology, Bioprospecting of brown seaweeds for biotechnological applications: Phlorotannin actions in inflammation and allergy network. Trends Food Sci. Technol..

[64] Jung H.A., Jin S.E., Ahn B.R., Lee C.M., Choi J.S. (2013). Anti-inflammatory activity of edible brown alga *Eisenia bicyclis* and its constituents fucosterol and phlorotannins in LPS-stimulated RAW264.7 macrophages. Food Chem. Toxicol..

[65] Barbosa M., Lopes G., Ferreres F., Andrade P.B., Pereira D.M., Gil-Izquierdo Á., Valentão P. (2017). Phlorotannin extracts from Fucales: Marine polyphenols as bioregulators engaged in inflammation-related mediators and enzymes. Algal Res..

[66] Kim M.-M., Kim S.-K. (2010). Effect of phloroglucinol on oxidative stress and inflammation. Food Chem. Toxicol..

[67] Joung E.J., Lee M.S., Choi J.W., Kim J.S., Shin T., Jung B.M., Kim J.I., Kim H.R. (2012). Anti-inflammatory effects of phlorofucofuroeckol B-rich ethyl acetate fraction obtained from *Myagropsis myagroides* on lipopolysaccharide-stimulated RAW 264.7 cells and mouse edema. Int. Immunopharmacol..

[68] Gonçalves-Fernández C., Sineiro J., Moreira R., Gualillo O. (2019). Extraction and characterization of phlorotannin-enriched fractions from the Atlantic seaweed *Bifurcaria bifurcata* and evaluation of their cytotoxic activity in murine cell line. J. Appl. Phycol..

[69] Nair D., Vanuopadath M., Balasubramanian A., Iyer A., Ganesh S., Anil A.N., Vikraman V., Pillai P., Bose C., Nair B.G. (2019). Phlorotannins from *Padina tetrastromatica*: Structural characterisation and functional studies. J. Appl. Phycol..

[70] Yu D.-K., Lee B., Kwon M., Yoon N., Shin T., Kim N.-G., Choi J.-S., Kim H.-R. (2015). Phlorofucofuroeckol B suppresses inflammatory responses by down-regulating nuclear factor κB activation via Akt, ERK, and JNK in LPS-stimulated microglial cells. Int. Immunopharmacol..

[71] Yang Y.-I., Shin H.-C., Kim S.H., Park W.-Y., Lee K.-T., Choi J.-H. (2012). 6,6′-Bieckol, isolated from marine alga *Ecklonia cava*, suppressed LPS-induced nitric oxide and PGE2 production and inflammatory cytokine expression in macrophages: The inhibition of NFκB. Int. Immunopharmacol..

[72] Hamed I., Özogul F., Özogul Y., Regenstein J.M. (2015). Marine bioactive compounds and their health benefits: A review. Compr. Rev. Food Sci. Food Saf..

[73] Bratchkova A., Kroumov A.D. (2020). Microalgae as producers of biologically active compounds with antibacterial, antiviral, antifungal, antialgal, antiprotozoal, antiparasitic and anticancer activity. Acta Microbiol. Bulg..

[74] Manganyi M.C., Ateba C.N.J.M. (2020). Untapped potentials of endophytic fungi: A review of novel bioactive compounds with biological applications. Microorganisms.

[75] Jose G.M. (2015). Biological Responses of Algal Derived Sulfated Polysaccharides: An Emphasis on Cancer Prophylaxis. Trends Biomater. Artif. Organs.

[76] Hussain E., Wang L.-J., Jiang B., Riaz S., Butt G.Y., Shi D.-Y. (2016). A review of the components of brown seaweeds as potential candidates in cancer therapy. RSC Adv..

[77] Meng W., Mu T., Sun H., Garcia-Vaquero M. (2021). Phlorotannins: A review of extraction methods, structural characteristics, bioactivities, bioavailability, and future trends. Algal Res..

[78] Ahn J.-H., Yang Y.-I., Lee K.-T., Choi J.-H. (2015). Dieckol, isolated from the edible brown algae *Ecklonia cava*, induces apoptosis of ovarian cancer cells and inhibits tumor xenograft growth. J. Cancer Res. Clin. Oncol..

[79] Kong C.-S., Kim J.-A., Yoon N.-Y., Kim S.-K. (2009). Induction of apoptosis by phloroglucinol derivative from *Ecklonia Cava* in MCF-7 human breast cancer cells. Food Chem. Toxicol..

[80] Kim R.-K., Suh Y., Yoo K.-C., Cui Y.-H., Hwang E., Kim H.-J., Kang J.-S., Kim M.-J., Lee Y.Y., Lee S.-J. (2015). Phloroglucinol suppresses metastatic ability of breast cancer cells by inhibition of epithelial-mesenchymal cell transition. Cancer Sci..

[81] Kang M.-H., Kim I.-H., Nam T.-J. (2014). Phloroglucinol induces apoptosis via apoptotic signaling pathways in HT-29 colon cancer cells. Oncol. Rep..

[82] Abdelhamid A., Lajili S., Elkaibi M.A., Ben Salem Y., Abdelhamid A., Muller C.D., Majdoub H., Kraiem J., Bouraoui A. (2019). Optimized Extraction, Preliminary Characterization and Evaluation of the in vitro Anticancer Activity of Phlorotannin-Rich Fraction from the Brown Seaweed, Cystoseira sedoides. J. Aquat. Food Prod. Technol..

[83] Sadeeshkumar V., Duraikannu A., Ravichandran S., Fredrick W.S., Sivaperumal R., Kodisundaram P. (2016). Protective effects of dieckol on N-nitrosodiethylamine induced hepatocarcinogenesis in rats. Biomed. Pharmacother..

[84] Antolovich M., Prenzler P.D., Patsalides E., McDonald S., Robards K. (2002). Methods for testing antioxidant activity. Analyst.

[85] Fubini B., Hubbard A. (2003). Reactive oxygen species (ROS) and reactive nitrogen species (RNS) generation by silica in inflammation and fibrosis. Free. Radic. Biol. Med..

[86] Nigam V., Sodhi J.S. (2014). Some medicinal plants with antioxidant activity—A review. Int. J. Pharm. Biol. Sci..

[87] Diniz do Nascimento L., De Moraes A.A.B., Da Costa K.S., Galúcio J.M.P., Taube P.S., Costa C.M.L., Cruz J.N., de Aguiar Andrade E.H., De Faria L.J.G. (2020). Bioactive Natural Compounds and Antioxidant Activity of Essential Oils from Spice Plants: New Findings and Potential Applications. Biomolecules.

[88] Chaves N., Santiago A., Alías J.C. (2020). Quantification of the Antioxidant Activity of Plant Extracts: Analysis of Sensitivity and Hierarchization Based on the Method Used. Antioxidants.

[89] Sathya R., Kanaga N., Sankar P., Jeeva S. (2017). Antioxidant properties of phlorotannins from brown seaweed *Cystoseira trinodis* (Forsskål) C. Agardh. Arab. J. Chem..

[90] Yotsu-Yamashita M., Kondo S., Segawa S., Lin Y.-C., Toyohara H., Ito H., Konoki K., Cho Y., Uchida T. (2013). Isolation and Structural Determination of Two Novel Phlorotannins from the Brown Alga *Ecklonia kurome* Okamura, and Their Radical Scavenging Activities. Marine Drugs.

[91] Ahn G.-N., Kim K.-N., Cha S.-H., Song C.-B., Lee J., Heo M.-S., Yeo I.-K., Lee N.-H., Jee Y.-H., Kim J.-S. (2007). Antioxidant activities of phlorotannins purified from *Ecklonia cava* on free radical scavenging using ESR and H_2_O_2_-mediated DNA damage. Eur. Food Res. Technol..

[92] Nakai M., Kageyama N., Nakahara K., Miki W. (2006). Phlorotannins as Radical Scavengers from the Extract of *Sargassum ringgoldianum*. Mar. Biotechnol..

[93] Boi V.N., Trang N.T.M., Cuong D.X., Ha H.T. (2020). Antioxidant Phlorotannin from Brown Algae *Sargassum dupplicatum*: Enzyme-assissted Extraction and Purification. World J. Food Sci. Technol..

[94] Lee J.-H., Ko J.-Y., Oh J.-Y., Kim E.-A., Kim C.-Y., Jeon Y.-J. (2015). Evaluation of phlorofucofuroeckol-A isolated from *Ecklonia cava* (Phaeophyta) on anti-lipid peroxidation in vitro and in vivo. Algae.

[95] Kang M.-C., Cha S.H., Wijesinghe W.A.J.P., Kang S.-M., Lee S.-H., Kim E.-A., Song C.B., Jeon Y.-J. (2013). Protective effect of marine algae phlorotannins against AAPH-induced oxidative stress in zebrafish embryo. Food Chem..

[96] Kang S.-M., Cha S.-H., Ko J.-Y., Kang M.-C., Kim D., Heo S.-J., Kim J.-S., Heu M.S., Kim Y.-T., Jung W.-K. (2012). Neuroprotective effects of phlorotannins isolated from a brown alga, *Ecklonia cava*, against H_2_O_2_-induced oxidative stress in murine hippocampal HT22 cells. Environ. Toxicol. Pharmacol..

[97] Kang X., Liang H., Luo Y., Li Z., He F., Han X., Zhang L. (2021). Anti-adipogenesis and metabolism-regulating effects of heat-inactivated *Streptococcus thermophilus* MN-ZLW-002. Lett. Appl. Microbiol..

[98] Blüher M. (2019). Obesity: Global epidemiology and pathogenesis. Nat. Rev. Endocrinol..

[99] Tsai Y.-C., Yang B.-C., Peng W.-H., Lee Y.-M., Yen M.-H., Cheng P.-Y. (2017). Heme oxygenase-1 mediates anti-adipogenesis effect of raspberry ketone in 3T3-L1 cells. Phytomedicine.

[100] Jung H.A., Jung H.J., Jeong H.Y., Kwon H.J., Ali M.Y., Choi J.S. (2014). Phlorotannins isolated from the edible brown alga *Ecklonia stolonifera* exert anti-adipogenic activity on 3T3-L1 adipocytes by downregulating C/EBPα and PPARγ. Fitoterapia.

[101] Karadeniz F., Ahn B.-N., Kim J., Seo Y., Jang M.-S., Nam K.-H., Kim M., Lee S.-H., Kong C.-S. (2015). Phlorotannins suppress adipogenesis in pre-adipocytes while enhancing osteoblastogenesis in pre-osteoblasts. Arch. Pharmacal Res..

[102] Seo Y.-J., Kim K.-J., Koh E.-J., Choi J., Lee B.-Y. (2017). Anti-adipogenesis mechanism of pterostilbene through the activation of heme oxygenase-1 in 3T3-L1 cells. Phytomedicine.

[103] Guo L., Li K., Kang J.S., Kang N.J., Son B.G., Choi Y.W. (2020). Strawberry fermentation with *Cordyceps militaris* has anti-adipogenesis activity. Food Biosci..

[104] Kong C.S., Kim H., Seo Y. (2015). Edible Brown Alga *Ecklonia cava* Derived Phlorotannin-Induced Anti-Adipogenic Activity in vitro. J. Food Biochem..

[105] Hu X., Tao N., Wang X., Xiao J., Wang M. (2016). Marine-derived bioactive compounds with anti-obesity effect: A review. J. Funct. Foods.

[106] Eom S.H., Lee S.H., Yoon N.Y., Jung W.K., Jeon Y.J., Kim S.K., Lee M.S., Kim Y.M. (2012). α-Glucosidase-and α-amylase-inhibitory activities of phlorotannins from *Eisenia bicyclis*. J. Sci. Food Agric..

[107] Lee S.-H., Yong L., Karadeniz F., Kim M.-M., Kim S.-K. (2009). α-Glucosidase and α-amylase inhibitory activities of phloroglucinal derivatives from edible marine brown alga, *Ecklonia cava*. J. Sci. Food Agric..

[108] Gheda S., Naby M.A., Mohamed T., Pereira L., Khamis A. (2021). Antidiabetic and antioxidant activity of phlorotannins extracted from the brown seaweed *Cystoseira compressa* in streptozotocin-induced diabetic rats. Environ. Sci. Pollut. Res..

[109] Catarino M.D., Silva A., Mateus N., Cardoso S.M. (2019). Optimization of phlorotannins extraction from *Fucus vesiculosus* and evaluation of their potential to prevent metabolic disorders. Marine Drugs.

[110] Ryu B., Jiang Y., Kim H.-S., Hyun J.-M., Lim S.-B., Li Y., Jeon Y.-J. (2018). Ishophloroglucin A, a novel phlorotannin for standardizing the anti-α-glucosidase activity of *Ishige okamurae*. Marine Drugs.

[111] You H.-N., Lee H.-A., Park M.-H., Lee J.-H., Han J.-S. (2015). Phlorofucofuroeckol A isolated from *Ecklonia cava* alleviates postprandial hyperglycemia in diabetic mice. Eur. J. Pharmacol..

[112] Kang M.-C., Wijesinghe W.A.J.P., Lee S.-H., Kang S.-M., Ko S.-C., Yang X., Kang N., Jeon B.-T., Kim J., Lee D.-H. (2013). Dieckol isolated from brown seaweed *Ecklonia cava* attenuates type II diabetes in db/db mouse model. Food Chem. Toxicol..

[113] Kim S.-K., Kong C.-S. (2010). Anti-adipogenic effect of dioxinodehydroeckol via AMPK activation in 3T3-L1 adipocytes. Chem.-Biol. Interact..

[114] Heo S.-J., Ko S.-C., Cha S.-H., Kang D.-H., Park H.-S., Choi Y.-U., Kim D., Jung W.-K., Jeon Y.-J. (2009). Effect of phlorotannins isolated from *Ecklonia cava* on melanogenesis and their protective effect against photo-oxidative stress induced by UV-B radiation. Toxicol. Vitr..

[115] Liu Y., Zhang D., Liu G.-M., Chen Q., Lu Z. (2019). Ameliorative effect of dieckol-enriched extraction from *Laminaria japonica* on hepatic steatosis induced by a high-fat diet via β-oxidation pathway in ICR mice. J. Funct. Foods.

[116] Ko S.-C., Lee M., Lee J.-H., Lee S.-H., Lim Y., Jeon Y.-J. (2013). Dieckol, a phlorotannin isolated from a brown seaweed, *Ecklonia cava*, inhibits adipogenesis through AMP-activated protein kinase (AMPK) activation in 3T3-L1 preadipocytes. Environ. Toxicol. Pharmacol..

[117] Filippini M., Baldisserotto A., Menotta S., Fedrizzi G., Rubini S., Gigliotti D., Valpiani G., Buzzi R., Manfredini S., Vertuani S. (2021). Heavy metals and potential risks in edible seaweed on the market in Italy. Chemosphere.

[118] Rajaram R., Rameshkumar S., Anandkumar A. (2020). Health risk assessment and potentiality of green seaweeds on bioaccumulation of trace elements along the Palk Bay coast, Southeastern India. Mar. Pollut. Bull..

[119] Chen Q., Pan X.-D., Huang B.-F., Han J.-L. (2018). Distribution of metals and metalloids in dried seaweeds and health risk to population in southeastern China. Sci. Rep..

[120] Cassani L., Gomez-Zavaglia A., Jimenez-Lopez C., Lourenço-Lopes C., Prieto M.A., Simal-Gandara J. (2020). Seaweed-based natural ingredients: Stability of phlorotannins during extraction, storage, passage through the gastrointestinal tract and potential incorporation into functional foods. Food Res. Int..

[121] Corona G., Ji Y., Anegboonlap P., Hotchkiss S., Gill C., Yaqoob P., Spencer J.P., Rowland I. (2016). Gastrointestinal modifications and bioavailability of brown seaweed phlorotannins and effects on inflammatory markers. Br. J. Nutr..

[122] Rajha H.N., Paule A., Aragonès G., Barbosa M., Caddeo C., Debs E., Dinkova R., Eckert G.P., Fontana A., Gebrayel P. (2022). Recent Advances in Research on Polyphenols: Effects on Microbiota, Metabolism, and Health. Mol. Nutr. Food Res..

[123] Rajan D.K., Mohan K., Zhang S., Ganesan A.R. (2021). Dieckol: A brown algal phlorotannin with biological potential. Biomed. Pharmacother..

[124] Michalak I., Chojnacka K. (2015). Algae as production systems of bioactive compounds. Eng. Life Sci..

[125] Stengel D.B., Connan S. (2015). Marine algae: A source of biomass for biotechnological applications. Natural Products from Marine Algae.

[126] Dang T.T., Bowyer M.C., Van Altena I.A., Scarlett C.J. (2018). Optimum conditions of microwave-assisted extraction for phenolic compounds and antioxidant capacity of the brown alga *Sargassum vestitum*. Sep. Sci. Technol..

[127] Kadam S.U., Tiwari B.K., O’Donnell C.P. (2013). Application of novel extraction technologies for bioactives from marine algae. J. Agric. Food Chem..

[128] Thiyagarasaiyar K., Goh B.-H., Jeon Y.-J., Yow Y.-Y. (2020). Algae metabolites in cosmeceutical: An overview of current applications and challenges. Mar. Drugs.

[129] Quitério E., Soares C., Ferraz R., Delerue-Matos C., Grosso C. (2021). Marine Health-Promoting Compounds: Recent Trends for Their Characterization and Human Applications. Foods.

[130] Corsetto P.A., Montorfano G., Zava S., Colombo I., Ingadottir B., Jonsdottir R., Sveinsdottir K., Rizzo A.M. (2020). Characterization of antioxidant potential of seaweed extracts for enrichment of convenience food. Antioxidants.

[131] Santos S.A., Félix R., Pais A., Rocha S.M., Silvestre A.J. (2019). The quest for phenolic compounds from macroalgae: A review of extraction and identification methodologies. Biomolecules.

[132] Brglez Mojzer E., Knez Hrnčič M., Škerget M., Knez Ž., Bren U. (2016). Polyphenols: Extraction methods, antioxidative action, bioavailability and anticarcinogenic effects. Molecules.

[133] Buedenbender L., Astone F.A., Tasdemir D. (2020). Bioactive molecular networking for mapping the antimicrobial constituents of the baltic brown alga *fucus vesiculosus*. Mar. Drugs.

[134] Al-Mola H.F. (2009). Antibacterial activity of crude extracts and phlorotannin isolated from the diatom *Cymbella* spp.. J. Pharm. Res.

[135] Kang M.-H., Kim I.-H., Nam T.-J. (2014). Phloroglucinol induces apoptosis through the regulation of insulin-like growth factor 1 receptor signaling pathways in human colon cancer HT-29 cells. Int. J. Oncol..

[136] Vizetto-Duarte C., Custódio L., Gangadhar K.N., Lago J.H., Dias C., Matos A.M., Neng N., Nogueira J.M., Barreira L., Albericio F. (2016). Isololiolide, a carotenoid metabolite isolated from the brown alga *Cystoseira tamariscifolia*, is cytotoxic and able to induce apoptosis in hepatocarcinoma cells through caspase-3 activation, decreased Bcl-2 levels, increased p53 expression and PARP cleavage. Phytomedicine.

[137] Kang K.A., Lee K.H., Chae S., Zhang R., Jung M.S., Ham Y.M., Baik J.S., Lee N.H., Hyun J.W. (2006). Cytoprotective effect of phloroglucinol on oxidative stress induced cell damage via catalase activation. J. Cell. Biochem..

[138] Kang K.A., Zhang R., Chae S., Lee S.J., Kim J., Kim J., Jeong J., Lee J., Shin T., Lee N.H. (2010). Phloroglucinol (1,3,5-trihydroxybenzene) protects against ionizing radiation-induced cell damage through inhibition of oxidative stress in vitro and in vivo. Chem.-Biol. Interact..

[139] Piao M.J., Zhang R., Lee N.H., Hyun J.W. (2012). Phloroglucinol Attenuates Ultraviolet B Radiation-Induced Matrix Metalloproteinase-1 Production in Human Keratinocytes via Inhibitory Actions against Mitogen-Activated Protein Kinases and Activator Protein-1. Photochem. Photobiol..

[140] Shrestha S., Johnston M.R., Zhang W., Smid S.D. (2021). A phlorotannin isolated from *Ecklonia radiata*, Dibenzodioxin-fucodiphloroethol, inhibits neurotoxicity and aggregation of β-amyloid. Phytomed. Plus.

[141] Ryu B., Ahn B.-N., Kang K.-H., Kim Y.-S., Li Y.-X., Kong C.-S., Kim S.-K., Kim D.G. (2015). Dioxinodehydroeckol protects human keratinocyte cells from UVB-induced apoptosis modulated by related genes Bax/Bcl-2 and caspase pathway. J. Photochem. Photobiol. B Biol..

[142] Li Y.X., Li Y., Je J.Y., Kim S.K. (2015). Dieckol as a novel anti-proliferative and anti-angiogenic agent and computational anti-angiogenic activity evaluation. Environ. Toxicol. Pharmacol..

[143] Khan F., Oh D., Chandika P., Jo D.M., Bamunarachchi N.I., Jung W.K., Kim Y.M. (2022). Inhibitory activities of phloroglucinol-chitosan nanoparticles on mono- and dual-species biofilms of *Candida albicans* and bacteria. Colloids Surf. B Biointerfaces.

